# A Subcircuit in the Suprachiasmatic Nucleus Generates Wakefulness

**DOI:** 10.1002/advs.202505131

**Published:** 2025-07-21

**Authors:** Qiang Liu, Sang Soo Lee, Jiali Xiong, Shahin Ahmadi, Benjamin J. Bell, Mehmet F. Keles, Mark N. Wu

**Affiliations:** ^1^ Department of Neurology Johns Hopkins University School of Medicine Baltimore MD 21205 USA; ^2^ Biochemistry Cellular and Molecular Biology Graduate Program Johns Hopkins University School of Medicine Baltimore MD 21205 USA; ^3^ Solomon H. Snyder Department of Neuroscience Johns Hopkins University School of Medicine Baltimore MD 21205 USA

**Keywords:** arousal, circadian, mWAKE, SCN, sleep

## Abstract

The daily cycles of sleep and arousal are among the most prominent biological rhythms under circadian control. While the role of the suprachiasmatic nucleus (SCN) as the master circadian pacemaker is well‐established, the molecular and circuit mechanisms by which it regulates the rhythms of sleep and arousal remain poorly understood. It has previously shown that the *Drosophila* clock‐output molecule Wide Awake (WAKE) and its mammalian homolog mWAKE are expressed in fly clock neurons and dorsomedial hypothalamus neurons that promote arousal. Here, it is investigated whether mWAKE labels an arousal‐promoting SCN subcircuit in mice. Broad chemogenetic activation and inhibition of mWAKE^+^ cells lead to a marked increase in wakefulness and a stupor‐like phenotype, respectively. Optogenetic activation specifically of mWAKE‐expressing neurons in the SCN (SCN^mWAKE^ neurons) promotes arousal, while genetic knockout of mWAKE or impairment of CLOCK function in these neurons enhances wakefulness selectively at night. The latter phenotype corresponds to a specific increase in nighttime spiking of SCN^mWAKE^ neurons in *mWake* mutants. At the circuit level, SCN^mWAKE^ neuron signaling to the subparaventricular zone (SPZ) yields a stronger arousal phenotype. Together, these findings suggest that the genetic mechanisms regulating circadian control of sleep and arousal are evolutionarily conserved and define a clock‐regulated neural network important for rhythmic arousal.

## Graphical abstract

This study identifies a sub‐circuit within the circadian pacemaker suprachiasmatic nucleus (SCN) that promotes arousal. This sub‐circuit is labeled by the clock output molecule mWAKE, which suppresses its excitability in a time‐dependent manner. mWAKE‐expressing cells in the SCN (SCN^mWAKE^ cells) project to the subparaventricular zone (SPZ) to mediate their effects on arousal. Together, these findings reveal a circadian‐regulated pathway that dynamically balances sleep and arousal.

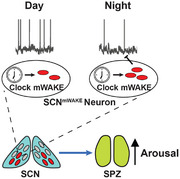

## 1 Introduction

More than 5 decades ago, the suprachiasmatic nucleus (SCN) was determined to house the master circadian pacemaker that coordinates nearly all daily biological rhythms in mammals.^[^ ^1^, ^2^, ^3^ ^]^ Sleep and arousal states are fundamental biological rhythms tightly regulated by the circadian clock. However, the molecular and circuit mechanisms by which SCN neurons mediate the cycling of sleep and arousal remain poorly understood.

The SCN can be broadly divided into core and shell subregions. The core subregion, densely populated by vasoactive intestinal peptide (VIP) and gastrin‐releasing peptide (GRP)‐expressing neurons, integrates light signals received via the retinohypothalamic tract, a process vital for aligning circadian rhythms with environmental light cycles.^[^ ^4^, ^5^, ^6^, ^7^ ^]^ GRP cells in the SCN (SCN^GRP^) are involved in light‐dependent resetting and collaborate with VIP neurons in the SCN (SCN^VIP^) to synchronize internal cellular rhythms across this brain region.^[^ ^8^, ^9^ ^]^ In contrast, the shell subregion, which contains arginine vasopressin (AVP) neurons (SCN^AVP^), generates self‐sustained circadian oscillations and orchestrates rhythmic output signals.^[^ ^10^, ^11^, ^12^ ^]^

While it is widely acknowledged that the SCN plays a critical role in regulating rhythms of sleep and wakefulness, the distinct subcircuits within the SCN required for this process are less clear. A few studies have shown that different SCN neurons exert distinct effects on sleep and arousal. For example, SCN^AVP^ neurons have no detectable impact on sleep or arousal, whereas activating SCN neurons expressing Neuromedin‐S (SCN^NMS^) promotes arousal, and stimulating a subset of SCN^VIP^ neurons selectively promotes nighttime sleep.^[^ ^6^, ^13^, ^14^ ^]^ In *Drosophila*, we have previously characterized the function of the clock‐output molecule WIDE AWAKE (WAKE) in regulating sleep onset and quality, by tuning the excitability of clock neurons.^[^ ^15^, ^16^ ^]^ The mammalian ortholog mWAKE (also named ANKFN1/Nmf9) is expressed in a variety of brain regions in mice, including the SCN, and has been shown to play a conserved role in modulating excitability in a cyclical manner to regulate various rhythmic behaviors.^[^ ^17^, ^18^ ^]^ Because our prior data have found that activating WAKE‐expressing neurons in flies and mice induces wakefulness,^[^ ^15^, ^16^, ^17^ ^]^ we hypothesized that mWAKE‐expressing neurons in the SCN (SCN^mWAKE^) constitute an arousal‐promoting subcircuit within the SCN.

Here, we show that chemogenetic activation or suppression of most or nearly all mWAKE^+^ cells induces marked changes in the level of arousal. Interestingly, optogenetic activation of SCN^mWAKE^ neurons is sufficient to induce wakefulness. We further show that knocking down mWAKE or impairing the function of the core clock molecule CLOCK in SCN^mWAKE^ cells significantly increases wakefulness, specifically at night. Finally, our data reveal that selective activation of the SCN^mWAKE^→SPZ (subparaventricular zone) circuit induces even stronger arousal than stimulation of all SCN^mWAKE^ neurons. Together, these findings refine our understanding of how circadian‐regulated circuitry dynamically balances sleep and arousal.

## 2 Results

### 2.1 mWAKE+ Neurons Play a Critical Role in Promoting Arousal

**Figure 1:**
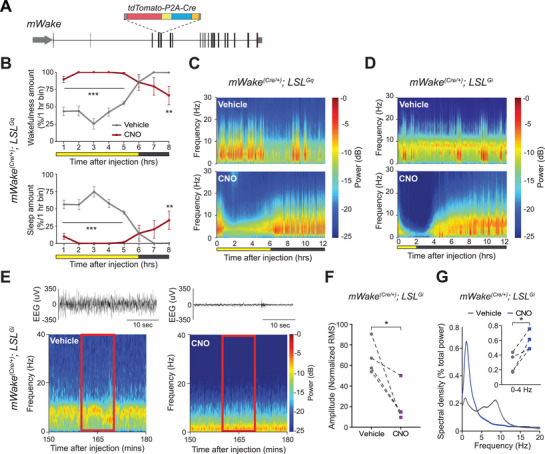
mWAKE labels a network critical for arousal. A) Schematic for the *mWake^(Cre)^* transgene showing the genomic locus of *mWake* and replacement of exon 5 with a *tdTomato‐P2A‐Cre*. B) Wakefulness (upper) and sleep (including both non‐Rapid Eye Movement/NREM and Rapid Eye Movement/REM) amount (% per 1 h bin) determined from EEG recordings following IP injection of either vehicle (gray) or 0.1 mg kg^−1^ CNO (red) into *mWake^(Cre/+)^; LSL^Gq^* mice at ZT6, n = 5. Yellow and black boxes indicate light and dark periods, respectively. Two‐way ANOVA with post‐hoc Sidak. ^**^ *p* < 0.01, ^***^ *p* < 0.001. C) Representative short‐time Fourier transform spectrograms from a *mWake^(Cre/+)^;LSL^Gq^* mouse, covering 12 h after IP injection of vehicle (top) or 0.1 mg kg^−1^ CNO (bottom) at ZT6, warmer colors indicate higher power of the frequency (y axis) at that specific time (x axis). D) Representative short‐time Fourier transform spectrograms of 12 h of recorded EEG activity from a *mWake^(Cre/+)^*; *LSL^Gi^* mouse after IP injection at ZT10 of vehicle alone (above) or 0.3 mg kg^−1^ CNO (below). E) Representative EEG traces for a 30 sec window (above) and short‐time Fourier transform spectrograms (below) from a *mWake^(Cre/+)^*; *LSL^Gi^* mouse starting 150 min (mins) after injection of vehicle (left) or 0.3 mg kg^−1^ CNO (right) at ZT10. The red box indicates the time window used for spectral and amplitude analysis in (F) and (G). F) EEG trace amplitude (plotted as normalized root mean square (RMS)) for *mWake^(Cre/+)^; LSL^Gi^* injected with vehicle (gray) or 0.3 mg kg^−1^ CNO (blue), n = 4. Unpaired Student's t‐test. ^*^ *p* < 0.05. G) Welch's power spectral density estimates as a percentage of total EEG power across 10 min, averaged across multiple EEG traces, for the animals described in (F). Inset shows a plot of delta‐band power as a percentage of total EEG power. Unpaired Student's t‐test. ^*^ *p* < 0.05. In this Figure and the following, error bars represent SEM. Full statistical details are provided in Table S1 (Supporting Information).

WAKE‐expressing neurons in *Drosophila* are arousal‐promoting, as are mWAKE^+^ neurons in the dorsomedial hypothalamus (DMH) in mice.^[^ ^15^, ^17^ ^]^ We wished to further investigate the role of mWAKE^+^ neurons in regulating arousal and wakefulness. To genetically target mWAKE^+^ neurons, while simultaneously creating a mutant allele, we previously generated transgenic mice (*mWake^(Cre^* ^)^) where exon 5 of *mWake* was replaced with a tdTomato‐P2A‐Cre cassette, which causes frameshifted nonsense mutations downstream, resulting in a loss‐of‐function allele of *mWake* (**Figure** **1**A). We hypothesized that, on balance, mWAKE broadly labels arousal‐promoting neurons. To test this hypothesis, we performed chemogenetic activation of most or nearly all mWAKE^+^ neurons. To do this, we crossed *mWake^(Cre/+)^* mice with a transgenic mouse line expressing DREADD‐hM3Dq under Cre‐dependent control (LSL^Gq^) to generate *mWake^(Cre/+)^; LSL^Gq^* progeny; these animals were injected with low‐dose clozapine N‐oxide (CNO) or vehicle control at Zeitgeber Time 6 (ZT6). DREADD‐Gq activation of most or all mWAKE^+^ neurons induced intense wakefulness with a dramatic reduction of sleep for ≈6 h after CNO injection (Figure 1B,C; Figure S1A; Table S1, Supporting Information). Next, we asked whether chemogenetically inhibiting most or nearly all mWAKE^+^ neurons would impair arousal. We crossed transgenic mice expressing Cre‐dependent DREADD‐hM4Di (*LSL^Gi^*) with *mWake^(Cre/+)^* mice to generate *mWake^(Cre/+)^; LSL^Gi^* progeny and injected CNO at ZT10. Electroencephalography (EEG) data demonstrated a marked reduction in signal amplitude, with slow frequency waveforms on EEG following injection of CNO, but not vehicle control (Figure 1D–G; Figure S1B; Table S1, Supporting Information). One CNO‐treated animal died >24 h after CNO injection. These phenotypes were suggestive of a stupor‐like state, rather than sleep, since the animals exhibited minimal to no responsiveness to gentle touch. Moreover, the low‐amplitude EEG waveforms were inconsistent with sleep (Figure S1C, Supporting Information); thus, the elevated delta power observed in the EEG signal likely reflects pathological, rather than sleep‐related, changes in cortical activity. For comparison, we assessed the EEG effects of chemogenetically silencing arousal‐promoting HDC^+^ (histamine decarboxylase) neurons, by repeating these experiments with *Hdc^(Cre/+)^; LSL^Gi^* mice. CNO treatment of these mice led to no observable differences in EEG spectral power and amplitude (Figure S1D; Figure S1G; Table S1, Supporting Information). Together, these findings suggest that the mWAKE^+^ network plays a crucial role in promoting arousal.

### 2.2 mWAKE Neurons in the SCN Promote Wakefulness

**Figure 2:**
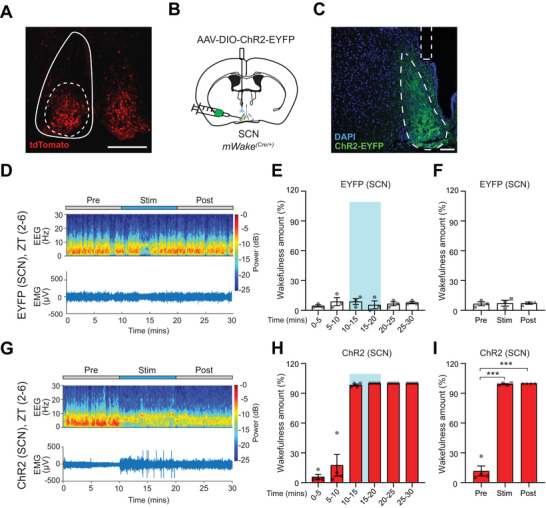
Optogenetically activating SCN^mWAKE^ neurons induces wakefulness. A) Native tdTomato fluorescence in the SCN of a *mWake^(Cre/+)^* mouse (solid and dashed lines denote SCN and core region, respectively). Scale bar represents 200 µm. B) Schematic showing unilateral injections of AAV‐DIO‐ChR2‐EYFP virus and optical fiber implantation into the SCN of an *mWake^(Cre/+)^* mouse. C) Representative confocal image of EYFP native fluorescence, DAPI signal, and optical fiber implantation in SCN for the mouse shown in (B). Dashed lines denote SCN and the fiber tract. Scale bar represents 100 µm. D) Representative short‐time Fourier transform spectrogram of EEG activity (above) and plot of EMG amplitude (below) across 10 min before (“Pre”), 10 min during (“Stim”), and 10 min after (“Post”) 10 Hz optogenetic stimulation of SCN^mWAKE^ neurons in control mice injected with AAV‐DIO‐EYFP virus into the SCN (EYFP (SCN), n = 3). Power density is depicted using a color scale and is decomposed by frequency on the y‐axis and time on the x‐axis. E) Wakefulness plotted as % time in 5 min bins. Optogenetic stimulation indicated by light blue box. % wakefulness for the 10 min before, during, and after blue LED stimulation of SCN^mWAKE^ neurons in animals described in (D). F) Average wakefulness as % time over the 10 min before (“Pre”), 10 min during (“Stim”), and 10 min after (“Post”) optogenetic stimulation of SCN^mWAKE^ neurons in control mice described in (D and E) (n = 3). One‐way ANOVA with post‐hoc Tukey. G) Representative short‐time Fourier transform spectrogram of EEG activity (above) and plot of EMG amplitude (below) across 10 min before (“Pre”), 10 min during (“Stim”), and 10 min after (“Post”) 10 Hz optogenetic stimulation of SCN^mWAKE^ neurons in animals injected with AAV‐DIO‐ChR2‐EYFP virus into the SCN (ChR2 (SCN), n = 4). Power density is depicted using a color scale and is decomposed by frequency on the y‐axis and time on the x‐axis. H) Wakefulness plotted as % time in 5 min bins. Optogenetic stimulation indicated by light blue box. % wakefulness for the 10 min before, during, and after optogenetic stimulation of SCN^mWAKE^ neurons in mice described in (G). I) Average wakefulness as % time over the 10 min before (“Pre”), 10 min during (“Stim”), and 10 min after (“Post”) optogenetic stimulation of SCN^mWAKE^ neurons in animals injected with AAV‐DIO‐ChR2‐EYFP virus into the SCN described in (G and H) (n = 4). One‐way ANOVA with post‐hoc Tukey. ^***^ *p* < 0.001.

Similar to the expression of WAKE in fly clock neurons, mWAKE is highly enriched in the SCN in mice. mWAKE does not label a specific sub‐cluster of SCN neurons, but instead is expressed across multiple SCN clusters (e.g., VIP‐, GRP‐, or prokineticin2/Prok2‐expressing cells).^[^ ^19^ ^]^ Because our data suggest that mWAKE^+^ neurons generally promote arousal, we hypothesized that mWAKE^+^ SCN neurons play a similar role. As previously described, mWAKE is expressed in the core region of the SCN (**Figure** **2**A).^[^ ^15^, ^19^ ^]^ To address the function of SCN^mWAKE^ neurons in regulating sleep or wakefulness, we unilaterally injected AAV‐DIO‐ChR2‐EYFP virus or a control virus into the SCN of *mWake^(Cre/+)^* mice (Figure 2B,C; Figure S2A; Table S2, Supporting Information). We then performed optogenetic stimulation of the SCN, while simultaneously recording EEG data. We first examined whether this manipulation would increase wakefulness during the daytime (when mice exhibit increased sleep). No effects on wakefulness were observed with optogenetic stimulation in control animals with injections of AAV‐DIO‐EYFP virus (Figure 2D–F; Table S1, Supporting Information). In contrast, optogenetic activation of SCN^mWAKE^ neurons led to a significant increase in wakefulness during stimulation, which persisted afterwards for ≈30 min (Figure 2G–I; Figure S2B,C; Table S1, Supporting Information). We next assessed whether a similar arousal‐promoting effect was observed if optogenetic activation of SCN^mWAKE^ neurons was performed during the nighttime. Stimulating SCN^mWAKE^ neurons during the active phase of the mice led to an increase in wakefulness (Figure S2D–F; Table S1, Supporting Information). These data suggest that activating SCN^mWAKE^ neurons enhances arousal.

### 2.3 mWAKE and CLOCK are Required in SCN^mWAKE^ Neurons for Generating Rhythmic Arousal

**Figure 3:**
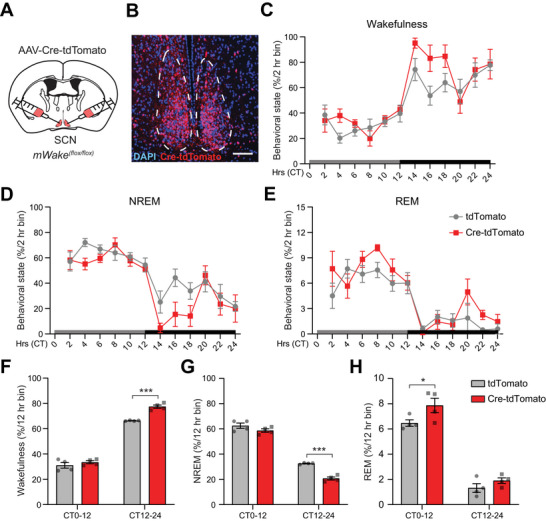
*mWAKE* knockdown in the SCN promotes wakefulness specifically at night. A) Schematic showing bilateral injections of AAV‐Cre‐P2A‐tdTomato virus into the SCN of an *mWake^(flox/flox)^* mouse. B) Representative confocal image of tdTomato native fluorescence and DAPI signal in SCN (dashed outline) for the mouse shown in (A), dashed lines denote SCN. Scale bar represents 50 µm. C–E) Behavioral state (Wakefulness (C), NREM (D), REM (E)) determined by EEG recordings (% per 2 h bin) for *mWake^(flox/flox)^* animals injected with AAV‐Cre‐P2A‐tdTomato (Cre‐tdTomato, n = 4, red) versus AAV‐tdTomato (tdTomato, n = 4, gray) under DD condition. Gray and black boxes indicate subjective day and subjective night, respectively. CT represents circadian time. F–H) Behavioral state (Wakefulness (F), NREM (G), REM (H)) determined by EEG recordings (% per 12 h bin) for the animals described in (C‐E). Two‐way ANOVA with post‐hoc Sidak. ^*^ *p* < 0.05, ^***^ *p* < 0.001.

Because mWAKE is a clock‐output molecule, we next investigated whether mWAKE functions in SCN^mWAKE^ cells to regulate arousal in a time‐dependent manner. To test this possibility, we performed conditional knockout of mWAKE by bilateral injection of AAV‐Cre‐tdTomato virus into the SCN of *mWake^(flox/flox)^* mice (**Figure** **3**A,B; Figure S3A; Table S2, Supporting Information), as we did previously for the DMH and lateral amygdala.^[^ ^17^, ^18^ ^]^ To confirm the efficacy of *mWake* knockdown following viral Cre injection, we performed RNAscope in situ hybridization, which revealed a substantial reduction in *mWake* transcript in the SCN (Figure S3B,C; Table S1, Supporting Information), likely due to nonsense‐mediated decay. We then assayed vigilance states by EEG recordings. Knockdown of *mWake* in the SCN significantly increased wakefulness and reduced NREM sleep during the subjective nighttime, but not the subjective daytime, compared to sham‐injected controls, in constant darkness (DD) (Figure 3C–G; Table S1, Supporting Information). Interestingly, REM sleep was slightly increased during the daytime in the conditional knockout animals (Figure 3E,H; Table S1, Supporting Information). A similar increase in wakefulness and reduction in NREM sleep during the nighttime was observed under light‐dark conditions (LD) (Figure S4A–F; Table S1, Supporting Information). These findings suggest that mWAKE acts in SCN neurons to inhibit arousal selectively at night.

**Figure 4:**
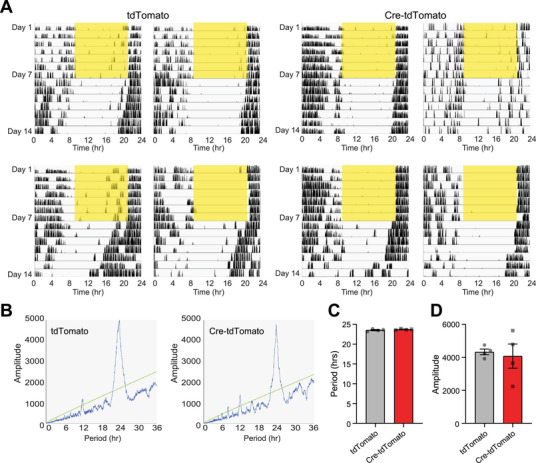
mWAKE in the SCN is not required for circadian rhythms. A) Wheel‐running traces of *mWake^(flox/flox^* ^)^ mice injected with AAV‐tdTomato (tdTomato) (left) or AAV‐Cre‐tdTomato (Cre‐tdTomato) (right) virus into the SCN (n = 4 each). 7 days of LD and 7 days of DD data are shown, with yellow bars indicating 12 h light periods. X‐axis represents time in hour. B) Representative chi‐squared periodograms for the mice described in (A). C and D) Average period length (C) and circadian rhythm amplitude (D) for the “tdTomato” (gray) and “Cre‐tdTomato” (red) animals described in (A). Unpaired Student's t‐test.

Next, we asked whether mWAKE expression in the SCN was required for circadian rhythms. A previous study using a different constitutive loss‐of‐function *mWake* allele (*Nmf9*) reported a mild but significant decrease in period length.^[^ ^20^ ^]^ First, we assessed period length and rhythm strength of locomotor rhythms in constitutive *mWake* knockout mice, using a wheel‐running assay. No change in period length was observed, while rhythm strength showed a mild, but not significant, reduction in *mWake^(‐/‐)^* mice, compared to littermate controls (Figure S5A–D; Table S1, Supporting Information). The slight difference between *mWake^(‐/‐)^* and *Nmf9^(‐/‐)^* animal phenotypes could be attributed to sex differences between males and females.^[^ ^20^ ^]^ To specifically assess the role of mWAKE in the SCN on circadian rhythms, we assessed period length and rhythm strength in mice with selective knockout of mWAKE in the SCN (Figure 3A,B; Figure S3A; Table S2, Supporting Information). This manipulation had no significant impact on circadian period length or rhythm strength (**Figure** **4**A–D; Table S1, Supporting Information). We next asked whether molecular clock rhythms were affected in these animals. To assess this, we examined expression of PER2 (a core clock molecule) at CT1 (Circadian Time 1) and CT13. mWAKE knockout in the SCN did not alter PER2 day/night cycling, compared to controls (Figure S5E,F; Table S1, Supporting Information), suggesting that molecular clock rhythms remain intact following this manipulation. Thus, in contrast to its role in regulating arousal, these findings suggest that mWAKE expression in the SCN is not essential for maintaining robust circadian rhythms.

**Figure 5:**
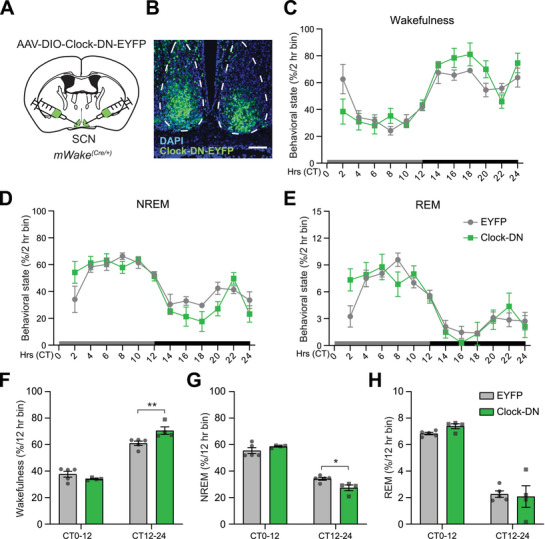
Disrupting Clock function in SCN^mWAKE^ neurons increases nighttime wakefulness. A) Schematic showing bilateral injections of AAV‐DIO‐Clock‐DN‐EYFP virus into the SCN of an *mWake^(Cre/+)^* mouse. B) Representative confocal image of EYFP native fluorescence and DAPI signal in SCN (dashed outline) for the mouse shown in (A), dashed lines denote SCN. Scale bar represents 50 µm. C–E) Behavioral state (Wakefulness (C), NREM (D), REM (E)) determined by EEG recordings (% per 2 h bin) for *mWake^(Cre/+)^* animals injected with AAV‐DIO‐Clock‐DN (Clock‐DN, green) (n = 4) versus AAV‐DIO‐EYFP (EYFP, gray) (n = 5) under DD condition. Gray and black boxes indicate subjective day and subjective night, respectively. CT denotes circadian time. F–H) Behavioral state (Wakefulness (F), NREM (G), REM (H)) determined by EEG recordings (% per 12 h bin) for animals described in (C‐E). Two‐way ANOVA with post‐hoc Sidak. ^*^ *p* < 0.05, ^**^ *p* < 0.01.

We have previously shown that rhythms of WAKE/mWAKE expression are dependent on the core circadian oscillator.^[^ ^15^, ^16^, ^17^, ^18^ ^]^ Thus, we investigated whether CLOCK function in SCN^mWAKE^ neurons is also required for the rhythmic regulation of arousal. To test this hypothesis, we expressed a dominant‐negative CLOCK construct in SCN^mWAKE^ neurons, by bilaterally injecting AAV‐DIO‐Clock‐DN‐EYFP virus^[^ ^18^ ^]^ into the SCN of *mWake^(Cre/+)^* animals (**Figure** **5**A,B; Figure S6A; Table S2, Supporting Information). As predicted, impairing CLOCK function in SCN^mWAKE^ neurons produced a phenotype similar to that seen with mWAKE conditional knockout in the SCN; wakefulness was enhanced and NREM sleep was reduced during the night, compared to control animals in both DD and LD (Figure 5C–H; Figure S6B–G; Table S1, Supporting Information). We next asked whether molecular and behavioral rhythms were affected in these animals. To investigate this, we first examined PER2 expression during subjective day versus subjective night. In control mice injected with AAV‐DIO‐EYFP, PER2 levels in the SCN exhibited robust day/night differences, with higher expression at CT13 compared to CT1 (Figure S7A,B; Table S1, Supporting Information) in both mWAKE^+^ and mWAKE^−^ cells. In contrast, mice expressing Clock‐DN in SCN^mWAKE^ cells showed a selective loss of PER2 day/night cycling in mWAKE^+^ cells, while mWAKE^−^ cells maintained significant day/night differences (Figure S7A,C; Table S1, Supporting Information). These results demonstrate both the efficiency and cell‐type specificity of the Clock‐DN virus in impairing core clock function. In terms of behavioral rhythms in these animals, neither period length nor locomotor activity rhythm strength was affected with Clock‐DN expression in SCN^mWAKE^ neurons (Figure S7D–G; Table S1, Supporting Information). Taken together, these findings suggest that both mWAKE and CLOCK in SCN are required in SCN^mWAKE^ neurons to regulate arousal in a time‐dependent manner.

### 2.4 mWAKE Suppresses SCN Firing at Night

**Figure 6:**
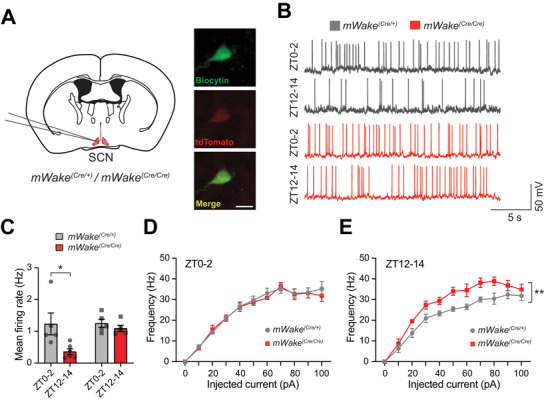
mWAKE inhibits the activity of SCN neurons at night. A) Schematic of whole‐cell patch‐clamp recordings from mWAKE^+^ neurons (tdTomato^+^ neurons in *mWake^(Cre/+)^* or *mWake^(Cre/Cre)^* mice), in the core region of the SCN (left). Representative images of a biocytin‐ and tdTomato‐labeled cell post‐recording (right). Scale bar denotes 25 µm. B) Representative membrane potential traces from whole‐cell patch clamp recordings of SCN^mWAKE^ neurons from *mWake^(Cre/+)^* (gray, top) and *mWake^(Cre/Cre)^* (red, bottom) slices at ZT0‐2 and ZT12‐14. C) Spontaneous mean firing rate for SCN^mWAKE^ neurons at ZT0‐2 and ZT12‐14 from *mWake^(Cre/+)^* (n = 5; 21 and n = 22 cells from 5 animals, gray) versus *mWake^(Cre/Cre)^* (n = 6 and 5; 24 and n = 20 cells from 5–6 animals, red) mice. ^*^ *p* < 0.05. D and E) Frequency‐current (*f*‐*I*) curves for SCN^mWAKE^ neurons from *mWake^(Cre/+)^* (gray) versus *mWake^(Cre/Cre)^* (red) mice at ZT0‐2 (n = 5; 21 and 19 cells from 5 animals) (D) or ZT12‐14 (n = 6 and 5; 19 and 13 cells from 5–6 animals) (E). Two‐way ANOVA with post‐hoc Sidak. ^**^ *p* < 0.01.

What underlying mechanism accounts for the night‐specific behavioral effects of mWAKE knockout in the SCN? In *Drosophila*, we previously showed that loss of WAKE leads to an increase in clock neuron spiking, specifically at night, which led to loss of daily rhythms of their activity.^[^ ^15^ ^]^ Moreover, we also demonstrated that DMH neurons in *mWake* mutants also exhibit a selective increase in firing rate at night.^[^ ^17^ ^]^ We thus asked whether *mWake* knockout would cause a similar phenotype in SCN^mWAKE^ neurons. Previous work has shown that, in general, SCN neuron spiking frequency is greater during the day versus the night.^[^ ^21^, ^22^, ^23^, ^24^ ^]^ We performed whole‐cell patch‐clamp slice recordings and first found, as expected, that the firing of SCN^mWAKE^ neurons in control *mWake^(Cre/+)^* mice followed a similar pattern (**Figure** **6**A–C; Table S1, Supporting Information). Next, we repeated these experiments in *mWake^(Cre/Cre^* ^)^ mutants and found a loss of cycling of spiking frequency in SCN^mWAKE^ neurons, due to a selective increase in spiking frequency at night (Figure 6B,C; Table S1, Supporting Information). The effects of *mWake* knockout on SCN^mWAKE^ activity are likely cell‐autonomous, as the intrinsic excitability of SCN^mWAKE^ neurons in these mutants was similarly increased during the night, but not the day (Figure 6D,E; Table S1, Supporting Information). These data suggest that, similar to its function in the DMH and LA, mWAKE suppresses neuronal excitability and activity in the SCN at night.

### 2.5 SCN^mWAKE^ Neurons Promote Arousal by Signaling to the SPZ

**Figure 7:**
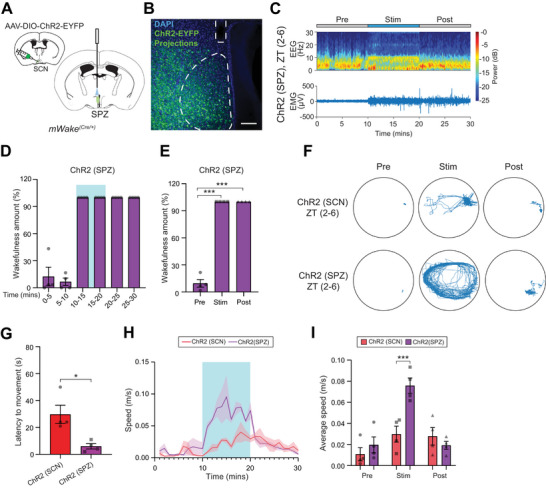
Optogenetic activation of the SCN^mWAKE^→SPZ circuit induces arousal. A) Schematic showing unilateral injections of AAV‐DIO‐ChR2‐EYFP virus into the SCN and optical fiber implantation into SPZ of an *mWake^(Cre/+)^* mouse. B) Representative confocal image of EYFP native fluorescence and DAPI signal, as well as location of optical fiber, in SPZ for the mouse shown in (A). Dashed lines denote SPZ and the fiber tract. Scale bar represents 100 µm. C) Representative short‐time Fourier transform spectrogram of EEG activity (above) and plot of EMG amplitude (below) across 10 min before (“Pre”), 10 min during (“Stim”), and 10 min after (“Post”) 10 Hz optogenetic stimulation of SPZ region using the mice described in (A and B). Power density is depicted using a color scale and is decomposed by frequency on the y‐axis and time on the x‐axis. D) Wakefulness, as determined by EEG, plotted as % time in 5 min bins for *mWake^(Cre/+)^* mice shown in (A). Optogenetic stimulation indicated by light blue box. % wakefulness for the 10 min before, during, and after optogenetic stimulation of SPZ region in mice described in (A‐C) (n = 4). E) Average wakefulness as % time over the 10 min before (“Pre”), 10 min during (“Stim”), and 10 min after (“Post”) optogenetic stimulation of SPZ region in mice described in (A‐D) (n = 4). One‐way ANOVA with post‐hoc Tukey. ^***^ *p* < 0.001. F) Representative locomotor activity tracks for the 10 min periods before (“Pre”), during (“Stim”), and after (“Post”) optogenetic activation of SCN^mWAKE^ neurons or downstream SPZ region. G) Latency to movement from NREM sleep following optogenetic activation of SCN^mWAKE^ neurons (red) or SCN^mWAKE^→SPZ circuit (purple) (n = 4 for both). Unpaired Student's t‐test. ^*^ *p* < 0.05. H) Average speed (m/sec) plotted per 1 min bins for optogenetic activation of SCN^mWAKE^ neurons (n = 4, red) or SCN^mWAKE^→SPZ projections (n = 4, purple). Period of optogenetic stimulation indicated by the light blue box. Shading denotes SEM. I) Mean speed (m/s) for the 10 min period before (“Pre”), during (“Stim”), or after (“Post”) optogenetic stimulation of SCN^mWAKE^ neurons (n = 4, red) or SCN^mWAKE^→SPZ projections (n = 4, purple); two‐way ANOVA with post‐hoc Sidak. ^***^ *p* < 0.001.

What are the circuit mechanisms by which an mWAKE‐expressing subset in the SCN promotes wakefulness? One potential downstream target for mediating this phenotype is the subparaventricular zone (SPZ), a key relay region downstream of the SCN that integrates circadian signals to regulate sleep and arousal states.^[^ ^13^, ^25^, ^26^, ^27^ ^]^ We thus hypothesized that SCN^mWAKE^ neurons signal to the SPZ network to promote wakefulness. To test this possibility, we unilaterally injected AAV‐DIO‐ChR2‐EYFP virus into the SCN and performed optogenetic stimulation of the terminals at SPZ during the daytime (ZT2‐6), while simultaneously recording EEG and video. Similar to the phenotype observed with activation of SCN^mWAKE^ cells, stimulating the SCN^mWAKE^ → SPZ pathway significantly promoted wakefulness (**Figure** **7**A–E; Figure S8A–C; Table S1, Supporting Information). Next, we compared the effects of activating the SCN^mWAKE^ → SPZ circuit versus directly stimulating SCN^mWAKE^ neurons. Interestingly, activating the SCN^mWAKE^ → SPZ circuit resulted in an overall stronger phenotype, as it reduced the time to transition from sleep to movement and increased movement speed compared to the activation of SCN^mWAKE^ neurons (Figure 7F–I; Video S1 and S2; Table S1, Supporting Information). Similar results were obtained when these experiments were performed during the nighttime (ZT14–18) (Figure S8D–F; Video S3 and S4; Table S1, Supporting Information). Together, these data suggest SCN^mWAKE^ neurons enhance arousal by signaling to a downstream SPZ region.

## 3 Discussion

Sleep and wakefulness are essential biological rhythms regulated by the circadian clock; however, the molecular and circuit mechanisms mediating this process remain unclear. The SCN functions as the circadian pacemaker in mammals, governing sleep and arousal rhythms, with distinct neuronal subpopulations playing specialized roles in sleep‐wake regulation. For instance, activating SCN^AVP^ neurons has no measurable effect on sleep or arousal, whereas activation of SCN^VIP^ neurons specifically enhances nighttime sleep.^[^ ^6^, ^13^ ^]^ In contrast, our findings demonstrate that optogenetic activation of SCN^mWAKE^ cells promotes wakefulness, a phenotype that is distinct from those other 2 major SCN clusters. Knockdown of *mWake* or impairing clock function in SCN^mWAKE^ neurons selectively increased wakefulness during the night. Consistent with this finding, SCN^mWAKE^ neurons in *mWake* mutants exhibit selectively increased excitability and spiking at night, further supporting the notion that SCN^mWAKE^ neurons promote wakefulness. Interestingly, however, SCN^mWAKE^ neurons normally exhibit higher daytime firing rates in wild‐type animals. Why would an arousal circuit fire more frequently during an animal's sleep phase? One possibility may relate to the nocturnal nature of these animals. Nocturnal animals experience fragmented sleep,^[^ ^28^ ^]^ which may provide an evolutionary advantage by lowering arousal threshold to facilitate responses to potential predators during the day. Thus, the SCN^mWAKE^ circuit may play a seemingly paradoxical role to promote arousal during the sleep phase, to support predator vigilance in nocturnal animals. In addition, why would mWAKE suppress arousal circuits during the night, when these animals are typically active? Similar to its role in the DMH,^[^ ^17^ ^]^ mWAKE may function as a clock‐dependent brake on arousal in the SCN, helping to fine‐tune and stabilize arousal levels.

While SCN^mWAKE^ neurons clearly modulate arousal, their contribution to the generation and maintenance of circadian rhythms appears to be limited. Specifically, we found that conditional knockout of mWAKE or expression of CLOCK‐DN in SCN^mWAKE^ neurons did not alter free‐running circadian period or rhythm strength. Although CLOCK‐DN expression disrupted local PER2 cycling within SCN^mWAKE^ neurons, this manipulation had no effect on overall behavioral rhythms (Figure S7A–G, Supporting Information), suggesting that these neurons are not essential for circadian regulation of locomotor activity. One potential explanation for this finding is that mWAKE expression is largely confined to GRP^+^ and VIP^+^ neurons in the SCN core, whereas the AVP^+^ shell region and broader NMS^+^ (neuromedin S‐expressing) populations are thought to be more critical for driving circadian behavior.^[^ ^11^, ^29^, ^30^ ^]^ Instead, SCN^mWAKE^ neurons may regulate circadian rhythms of arousal through time‐of‐day‐dependent modulation of neuronal excitability, functioning more as circadian output modulators than as rhythm generators.

Arousal can be behaviorally characterized as a state with increased locomotor activity and increased responsiveness to sensory or emotional stimuli.^[^ ^31^ ^]^ Interestingly, stimulation of SCN^mWAKE^→SPZ projections not only promotes rapid wakefulness, but also greater locomotor activity, compared to activation of SCN^mWAKE^ neurons. This finding suggests that the former manipulation leads to more heightened arousal. Why would activating one specific SCN^mWAKE^ projection yield a stronger phenotype than that seen with broad activation of all SCN^mWAKE^ neurons? One possible explanation is that SCN^mWAKE^ neurons project to multiple downstream targets, some of which may promote sleep or inhibit arousal/locomotion. Previous studies have demonstrated that the SCN exerts widespread influence over various brain regions involved in sleep regulation, including the ventrolateral preoptic area (VLPO).^[^ ^32^, ^33^, ^34^ ^]^ Thus, direct activation of SCN^mWAKE^ neurons may therefore engage both wake‐promoting and sleep‐promoting pathways, while the SCN^mWAKE^→SPZ circuit specifically activates arousal‐promoting mechanisms. This interpretation aligns with previous reports suggesting that the SPZ serves as a key relay integrating SCN signals to regulate arousal and locomotion.^[^ ^25^, ^27^ ^]^

At the molecular level, how mWAKE regulates neuronal excitability in the SCN remains an open question. Although mWAKE modulates BK currents to regulate rhythmic intrinsic excitability in the lateral amygdala,^[^ ^18^ ^]^ its functional targets within the SCN have not yet been identified. In *Drosophila*, WAKE also interacts with GABA_A_ receptors, upregulating its expression and enhancing its membrane localization.^[^ ^15^ ^]^ GABA_A_ receptors would represent an attractive molecular target for mWAKE in the SCN, given the abundant GABAergic signaling within the SCN and the essential role of GABA_A_ receptors in shaping SCN neuron excitability and network synchronization.

Our studies of mWAKE have revealed that circadian regulation of arousal and internal states extends beyond the suprachiasmatic nucleus (SCN) to other local brain regions, such as the DMH and lateral amygdala (LA).^[^ ^17^, ^18^ ^]^ In the LA, an oscillatory mechanism driven by mWAKE neurons modulates rhythmic sensory perception and internal states, highlighting a distributed network of cellular and behavioral rhythms.^[^ ^18^ ^]^ In the DMH, mWAKE functions as a clock‐dependent brake on arousal, ensuring the proper timing of wakefulness and preventing excessive activation during the inactive phase.^[^ ^17^ ^]^ DMH neurons likely receive circadian input through the subparaventricular zone (SPZ), but it remains unclear whether the SCN^mWAKE^→SPZ circuit signals directly to DMH neurons.^[^ ^25^, ^27^ ^]^ Future studies may further disentangle the downstream circuit mechanisms by which the master pacemaker SCN coordinates rhythms of sleep and wakefulness.

This study has several limitations that should be acknowledged. First, the interpretation of the findings from chemogenetic manipulation of mWAKE^+^ neurons using the *LSL^Gq^* or *LSL^Gi^* transgenic lines is complicated by the labeling of cells in many areas of the brain and throughout development. Moreover, in those experiments, we used DREADD‐Gi expression in *Hdc* ^+^ neurons as a control comparison. While *Hdc^Cre^* mice allow for access to arousal‐promoting neurons, this line likely expresses in fewer neurons compared to the *mWake^Cre^* line, limiting its utility as a direct control. Second, most behavioral experiments were conducted in male mice. Given emerging evidence that circadian output and arousal regulation may differ between sexes,^[^ ^35^, ^36^, ^37^ ^]^ this omission limits generalizability of findings. Lastly, we did not examine other potential projection targets of SCN^mWAKE^ neurons, such as the VLPO and DMH. Our previous single‐cell RNA sequencing data indicates that mWAKE expression in the SCN spans multiple clusters, such as those expressing VIP, GRP, Prok2 and NMS.^[^ ^19^ ^]^ This molecular and anatomical diversity implies that SCN^mWAKE^ neurons contribute to distinct SCN output pathways, each potentially regulating different aspects of sleep‐wake behavior depending on their projection targets. Future, more comprehensive circuit‐level analyses will be essential to fully define the broader network through which SCN^mWAKE^ neurons influence arousal.

## 4 Experimental Section

### Animals

All experimental protocols involving animals were reviewed and authorized by the Johns Hopkins Institutional Animal Care and Use Committee (IACUC). Mice were typically housed in groups, except under specific experimental conditions described below. Housing occurred in a shared facility with a 14‐hr light/10‐hr dark photoperiod (14:10 LD), followed by a minimum two‐week acclimation period to a 12‐hr light/12‐hr dark (12:12 LD) cycle in a separate satellite room prior to testing. Genotyping assays employed Taq‐Man rtPCR probes (Transnetyx). Wild‐type *C57BL/6J* mice (Jackson Laboratory, stock #000664) served as the genetic background, with all experimental strains backcrossed to this line at least seven generations. The study incorporated 5 genetically modified lines, which have been previously described: an mWAKE conditional allele (*mWake^flox^*),^[^ ^17^ ^]^ an mWAKE allele with a tdTomato‐P2A‐Cre cassette integrated into exon 5 (*mWake^Cre^*), ^[^ ^17^, ^18^ ^]^ *mWake* knockout (*Wake^−/−^)*,^[^ ^17^ ^]^ (*LSL^Gq^*),^[^ ^38^ ^]^ (*LSL^Gi^*)^[^ ^38^ ^]^ and (*Hdc^Cre^*).^[^ ^39^ ^]^

### Electroencephalography (EEG)—Surgery

Ale mice aged 8–10 weeks were anesthetized with isoflurane to induce surgical‐depth anesthesia. Following cranial fur removal, a midline incision was made along the anterior‐posterior axis of the skull. After exposing and cleaning the skull, a 3‐channel EEG headmount (Pinnacle Technology) was positioned 3 mm anterior to the bregma and secured using four manually drilled anchor screws. Bilateral EMG electrodes were implanted into the nuchal muscles, and the incision was sutured before finalizing headmount fixation with dental acrylic. All animals recovered for ≥ 2 weeks post‐surgery prior to experimental setups.

### EEG Recording

Sleep‐wake behavior was monitored using a tethered EEG/EMG system (Pinnacle Technology). Post‐recovery, mice were individually housed in cylindrical acrylic chambers (8‐inch diameter) with ad libitum access to food and water. Animals underwent a 5‐day acclimation period to tethering while maintained under a 12:12 LD cycle. Signals were amplified (100× gain), sampled at 400 Hz, and filtered (EEG: 0.5 Hz high‐pass; EMG: 10 Hz high‐pass) before digitization via Sirena Acquisition Software (Pinnacle Technology).

### Analysis

Sleep staging was conducted in 10 s epochs using Sirenia Sleep Software (Pinnacle Technology) by trained scorers blinded to experimental conditions. Epochs were classified as WAKE, NREM, or REM, with artifact‐contaminated intervals excluded from analysis. Animals exhibiting persistent signal noise or poor waveform quality were removed from datasets. Spectral analysis utilized custom MATLAB scripts (MathWorks) to compute Fast Fourier Transforms (FFT; 512–1024 points) with Welch's power spectral density estimation. Spectrograms were generated using short‐time Fourier transforms (30‐second Hann windows, 60% overlap).

### Stereotaxic Surgeries

Male mice aged 8 to 16 weeks were anesthetized to surgical depth using isoflurane, and the fur on the top of the head was completely removed. Each mouse was then secured in a Stoelting stereotaxic frame, and the microinjector tip was positioned at Bregma, with all coordinates set to zero. Small craniotomies (≈0.5 mm) were made to facilitate virus injections (50–100 nL) at a controlled rate of ≈50 nL min^−1^. Detailed information on injection coordinates, volumes, and virus sources can be found in Table S2 (Supporting Information). Following the injection, animals were single‐housed, and given at least two weeks to recover and allow for viral gene expression. EEG head mount and/or optogenetic fiber were implanted immediately after the virus injection. The accuracy of injection sites was confirmed through post‐hoc imaging.

### Designer Receptors Exclusively Activated by Designer Drugs (DREADDs)

DREADD receptors coupled to Gi or Gq were expressed in a Cre‐dependent fashion in mWAKE^+^ neurons of *mWake^(Cre/+)^* mice by crossing with a transgenic effector mouse *B6N;129‐CAG‐LSL‐HA‐hM4Di‐mCitrine* (a CAG‐promoter‐driven HA‐tagged hM4Di transgene and mCitrine fluorescent protein, both downstream of a Lox‐Stop‐Lox (LSL) sequence for Cre‐dependent expression; “*LSL^Gi^*”), or a B6N;129‐Tg(CAG‐CHRM3*‐mCitrine)1Ute/J line (a CAG‐promoter‐driven HA‐tagged hM3Dq transgene and mCitrine fluorescent protein, both downstream of a LSL sequence for Cre‐dependent expression, “*LSL^Gq^*”). Clozapine *N*‐oxide (CNO) (Sigma‐Aldrich) was prepared as a stock solution of 50 mg mL^−1^ in DMSO, and then freshly diluted to 0.1 mg mL^−1^ for *LSL^Gi^* and 0.05 mg mL^−1^ for *LSL^Gq^* in sterile PBS before intraperitoneal (IP) injection. Solution clarity was monitored throughout dosing, and the solution was warmed to 37 °C if precipitates were observed. Vehicle control was prepared as sterile saline + 0.01% DMSO. All injections occurred at the same ZT time within each experiment, and all animals (both males and females) were treated with vehicle or CNO each day in a cross‐over design, with ≥2 days recovery between experimental recording days. Each DREADD experiment included genetic controls (CNO injections in *mWake^(Cre/+)^*; *LSL^Gi^* or *mWake^(Cre/+)^*; *LSL^Gq^* animals).

### Optogenetics and Video Recording

AAV‐DIO‐ChR2‐EYFP or a control virus (AAV‐DIO‐EYFP) was unilaterally injected into the SCN of *mWake^(Cre/+)^* male mice, as detailed above and in Table S2 (Supporting Information). Following the injection, an optical fiber (200 µm O.D., 0.39 NA, RWD Life Science Inc.) housed within a ceramic ferrule was implanted toward the SCN or SPZ. An EEG head mount (Pinnacle Technology) was then secured to the skull using four screws, and dental acrylic was applied to affix both the ferrule and head mount in place. Mice were given at least two weeks to recover post‐surgery before being connected to the optogenetic stimulation system (Thorlabs, OGKL2) and EEG recording setup (Pinnacle Technology). After a minimum of four days of habituation, EEG recordings were conducted while optogenetic stimulation (470 nm, 4–6 mW, 10 Hz, 20% duty cycle) was applied for 10 min between ZT2 and ZT6. Animals were recorded for at least 1 h after stimulation. The duration to “Return to baseline” was defined as the time required for sleep to fall within the baseline (pre‐stimulation period) ± SEM range determined by EEG, within a 5 min window. A top‐mounted camera (DMK 22AUC03, LORE+ Technology) recorded video at 15 Hz. Following this, recordings were conducted using the same protocol between ZT14 and ZT18 under IR light.

### Video Analysis

To track animal position during optogenetic experiments, we used a computer vision‐based approach leveraging the YOLOv11 object detection model as previously described.^[^ ^40^ ^]^ The model was trained on a dataset of 160 manually annotated images, each containing a bounding box around the animal. These images were randomly sampled from video recordings of 8 different animals to enhance the model's generalizability. Training was conducted on two NVIDIA RTX 3090 GPUs for 200 epochs, with a batch size of 64 and an input resolution of 640 × 640 pixels. The final model achieved a mean average precision (mAP) of 0.94 across intersection over union (IoU) thresholds from 0.5 to 0.95 (mAP50–95). This trained model was then applied to behavioral video recordings to extract the animal's position, from which average speed was calculated in 1 min time bins.

### Immunostaining

Mice were deeply anesthetized and perfused with ice‐cold 1× PBS (1.1 mM KH₂PO₄, 155.2 mM NaCl, 3.0 mM Na₂HPO₄, pH 7.4), followed by 4% paraformaldehyde (PFA) in 1× PBS. Brains were dissected and post‐fixed overnight in 4% PFA at 4 °C. Coronal brain sections (40 µm) were cut using a vibratome (VT‐1200s, Leica), then washed twice in PBS containing 0.3% Triton X‐100 (PBS‐T). Sections were blocked in PBS‐T supplemented with 5% goat serum for 30 min at room temperature (RT), followed by incubation with primary antibodies diluted in the same blocking solution for 24 h at 4 °C. The following primary antibodies were used: rabbit anti‐PER2 (Millipore Sigma, AB2202, 1:500) and chicken anti‐RFP (Rockland, 600‐901‐379, 1:1000). After four washes in PBS‐T, sections were incubated overnight at 4 °C with fluorescent secondary antibodies diluted in PBS‐T with 5% goat serum. The following secondary antibodies were used: Alexa Fluor 647 goat anti‐rabbit (Invitrogen, A21244, 1:1000) for PER2 detection and Alexa Fluor 568 goat anti‐chicken (Invitrogen, A11041, 1:1000) for tdTomato detection. After four final washes in PBS‐T, sections were mounted with mounting medium with DAPI (Vector Labs, H‐1500‐10) and coverslips were added. Confocal imaging was performed using a Zeiss LSM700 microscope with 10× and 40× objectives.

### Quantification

PER2 immunoreactivity in the SCN was quantified using Fiji (ImageJ). Maximum intensity projections were generated from confocal z‐stacks of 40 µm slices, 3–5 slices per animal were used for the analysis. In animals with viral injections targeting the SCN, mWAKE+ cells were identified by co‐expression of tdTomato and EYFP. Cells lacking both tdTomato and EYFP signals were classified as mWAKE^−^.

### In Situ Hybridization

RNAscope in situ hybridization was performed using the RNAscope Multiplex Fluorescent Reagent Kit v2 (Advanced Cell Diagnostics, 323100) according to the manufacturer's instructions. Mice were anesthetized and perfused with ice‐cold 1× PBS followed by 4% PFA in 1× PBS. Brains were post‐fixed in 4% PFA for 24 h at 4 °C, then cryoprotected sequentially in 10%, 20%, and 30% sucrose solutions in PBS at 4 °C (1 day for 10% and 20%, 2 days for 30%) until they sank. Brains were embedded in Tissue‐Tek O.C.T. compound (VWR, 4583), frozen using dry ice, and stored at −80 °C. Coronal sections (13 µm) were collected using a cryostat, mounted on Superfrost Plus slides, and stored at −80 °C until use. RNAscope was then performed following the ACD protocol (320293). The following probe was used: mWake‐C1 (1057631‐C1). Signal amplification was achieved using Opal 690 dye (AKOYA Biosciences, NEL810001KT; 1:2000 dilution). Following RNAscope, immunostaining for tdTomato was performed on the same sections as described above. Sections were counterstained with DAPI (incubated for 30 seconds at RT), and mounted using ProLong Gold Antifade Mountant (Invitrogen, P36930) with coverslips. Confocal imaging was performed on a Zeiss LSM700 microscope using 40× objectives.

### Quantification

*mWake* RNAscope signal was quantified using Fiji. Maximum intensity projections were generated from 13 µm sections containing the SCN, 3 slices per animal were used for the analysis. The region of interest (ROI) within the SCN was manually defined based on tdTomato fluorescence in mice injected with AAV‐tdTomato or AAV‐Cre‐P2A‐tdTomato.

### Wheel‐Running Activity

Adult male mice (3–5 months old) were individually housed in cages equipped with a vertical running wheel (ActiMetrics) and had continuous access to food and water. Following a one‐week acclimatization period under a 12:12 h LD cycle, their wheel‐running activity was recorded for one week in LD conditions. This was followed by another week in constant darkness (DD) to assess free‐running activity. Wheel‐running data was collected in 1 min intervals and analyzed using ClockLab software (ActiMetrics). Period length and rhythm strength estimates were derived from 7 consecutive days of DD recordings. Two mutant animals were excluded due to markedly reduced wheel running, precluding reliable circadian rhythm analysis.

### Electrophysiological Recordings

Male mice aged 6 to 9 weeks were deeply anesthetized with isoflurane, after which their brains were rapidly extracted and dissected in an ice‐cold, oxygenated slicing solution (95% O₂, 5% CO₂) containing 2.5 mM KCl, 1.25 mM NaH₂PO₄, 2 mM MgSO₄, 2.5 mM CaCl₂, 248 mM sucrose, 26 mM NaHCO₃, and 10 mM glucose. Coronal brain slices (250 µm thick) were generated using a vibratome (VT‐1200s, Leica) and subsequently incubated in oxygenated artificial cerebrospinal fluid (ACSF: 124 mM NaCl, 2.5 mM KCl, 1.25 mM NaH₂PO₄, 2 mM MgSO₄, 2.5 mM CaCl₂, 26 mM NaHCO₃, and 10 mM glucose; 290–300 mOsm). The slices were maintained at 28 °C for 30 min before being transferred to room temperature for an additional hour. For electrophysiological recordings, the slices were placed in a recording chamber with continuous perfusion of oxygenated ACSF at room temperature. Cells of interest were identified using an upright microscope (BX51WI, Olympus) with infrared differential interference contrast (IR‐DIC) and native fluorescence imaging. Patch‐clamp recordings were performed using glass electrodes (5–8 MΩ) filled with an internal solution consisting of 130 mM K‐gluconate, 5 mM NaCl, 10 mM C₄H₈N₃O₅PNa₂, 1 mM MgCl₂, 0.2 mM EGTA, 10 mM HEPES, 2 mM MgATP, and 0.5 mM Na₂GTP (pH 7.2–7.3, 300 mOsm). Whole‐cell patch‐clamp recordings were acquired using a Multiclamp 700B amplifier (Molecular Devices), with data sampled at 20 kHz, low‐pass filtered at 2 kHz, and digitized using a Digidata 1440A (Molecular Devices).

### Statistical Analysis

Statistical analyses were conducted using Prism 8 (GraphPad). For comparisons between two groups that are normally distributed, unpaired Student's t‐tests, two‐tailed were used. For analyses involving multiple factors, two‐way ANOVAs were conducted for normally distributed data, with repeated measures applied when appropriate and post hoc Sidak tests for pairwise comparisons following ANOVA. Data are presented as mean ± SEM for all figures; sample size (n), *p* value and all statistical details are indicated in each figure legend and Table S1 (Supporting Information).

## Conflict of Interest

The authors declare no conflict of interest.

## Author Contributions

Q.L. and M.N.W. conceived the project. Q.L. performed mouse genetics, behavioral experiments, surgeries, imaging, electrophysiological and optogenetic experiments. B.B. performed DREADD experiments. Q. L, S.L, J.X, S. A, and M. K performed analyses. Q.L. and M.N.W. wrote the manuscript, with feedback from all authors.

## Supporting information



Supporting Information

Supplemental Table 1

Supplemental Video 1

Supplemental Video 2

Supplemental Video 3

Supplemental Video 4

## Data Availability

The data that support the findings of this study are available in the supplementary material of this article.
