# Supplementary material for: A Subcircuit in the Suprachiasmatic Nucleus Generates Wakefulness
Source: Adv Sci (Weinh). 2025 Jul 21;12(39):e05131. doi: 10.1002/advs.202505131 (PMC12533371; doi:10.1002/advs.202505131)
Supplement: Supplementary file 1 — Supporting Information [file ADVS-12-e05131-s005.pdf]

ADVANCED SCIENCE
Open Access

Supporting Information

for *Adv. Sci.*, DOI 10.1002/advs.202505131

A Subcircuit in the Suprachiasmatic Nucleus Generates Wakefulness

*Qiang Liu, Sang Soo Lee, Jiali Xiong, Shahin Ahmadi, Benjamin J. Bell, Mehmet F. Keles and Mark N. Wu**

A Subcircuit in the Suprachiasmatic Nucleus Generates Wakefulness

Qiang Liu[1], Sang Soo Lee[1], Jiali Xiong[2], Shahin Ahmadi[1], Benjamin J. Bell[1], Mehmet F. Keles[1], and Mark N. Wu[1,3,#]

[1]Department of Neurology, Johns Hopkins University School of Medicine, Baltimore, MD 21205

[2]Biochemistry, Cellular and Molecular Biology Graduate Program, Johns Hopkins University School of Medicine, Baltimore MD 21205

[3]Solomon H. Snyder Department of Neuroscience, Johns Hopkins University School of Medicine, Baltimore, MD 21205

[#]Correspondence should be addressed to M.N.W. (marknwu@jhmi.edu)

Figure S1. Chemogenetic inhibition of HDC⁺ neurons does not alter EEG spectral power and amplitude, related to Figure 1.

(A) Representative EEG (top) and EMG (bottom) traces from *mWake*$^{(Cre/+)}$; *LSL*^{Gq} mice following IP injection of vehicle (left) or 0.1 mg/kg CNO (right) at ZT6. Traces are from the same time window (ZT9-11) under both conditions. Vigilance states for vehicle condition (NREM, NREM, NREM for animals 1-3, respectively) and CNO condition (Wake, Wake, Wake for animals 1-3, respectively).

(B) Representative EEG (top) and EMG (bottom) traces from *mWake*$^{(Cre/+)}$; *LSL*^{Gi} mice following IP injection of vehicle (left) or 0.3 mg/kg CNO (right) at ZT10. Traces are from the same time window (ZT11-13) under both conditions. Vigilance states for vehicle condition (Wake, Wake, Wake for animals 1-3, respectively) and CNO condition (stupor-like, stupor-like, stupor-like for animals 1-3, respectively).

(C) Representative EEG (above) and EMG (below) traces of NREM, REM, and "stupor-like" states recorded from a *mWake*$^{(Cre/+)}$; *LSL*^{Gi} mouse.

(D) Representative short-time Fourier transform spectrograms of 12 hrs of recorded EEG activity from a *Hdc*$^{(Cre/+)}$*; LSL*^{Gi} mouse after IP injection at ZT10 of vehicle alone (above) or 1 mg/kg CNO (below) under LD condition. Yellow and black boxes indicate light and dark periods, respectively.

(E) Short-time Fourier transform spectrograms for a 30 min time window for an *Hdc*$^{(Cre/+)}$*;LSL*^{Gi} mouse starting 150 mins after injection of vehicle (left) or 1 mg/kg CNO (right) at ZT10. The red box indicates the time window used for spectral and amplitude analysis in (**F**) and (**G**).

(F) EEG trace amplitude (plotted as normalized root mean square (RMS)) for *Hdc*$^{(Cre/+)}$*; LSL*^{Gi} injected with vehicle (gray) or 1 mg/kg CNO (green), n=3. Unpaired Student's t-test.

(G) Welch's power spectral density estimates as a percentage of total EEG power across 10 min, averaged across multiple EEG traces for the animals described in (**F**). Inset shows a plot of delta-band power as a percentage of total EEG power. Unpaired Student's t-test.

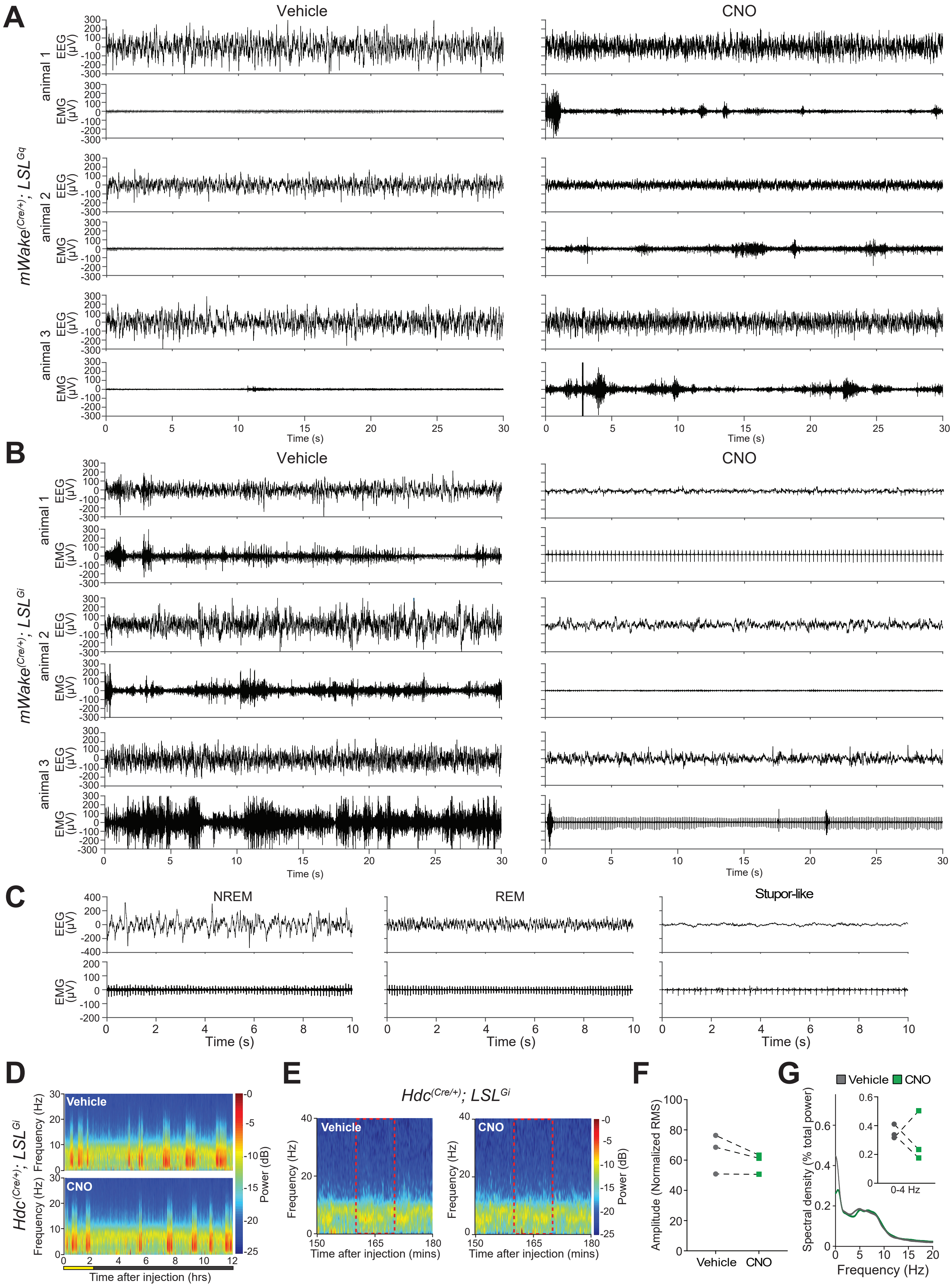
A
Vehicle
CNO
animal 1
animal 2
animal 3
EEG (µV)
EMG (µV)
mWake(Cre/+); LSLGq
Time (s)
B
Vehicle
CNO
mWake(Cre/+); LSLGi
Time (s)
C
NREM
REM
Stupor-like
Time (s)
D
Vehicle
CNO
Hdc(Cre/+); LSLGi
Frequency (Hz)
Power (dB)
Time after injection (hrs)
E
Hdc(Cre/+); LSLGi
Time after injection (mins)
F
Amplitude (Normalized RMS)
Vehicle
CNO
G
Vehicle
CNO
Spectral density (% total power)
0-4 Hz
Frequency (Hz)

Figure S1

Figure S2. Additional data related to Figure 2.

(A) Confocal images showing native EYFP fluorescence, DAPI staining, and optical fiber placement in the SCN of *mWake*$^{(Cre/+)}$ mice following targeted injection of AAV-DIO-ChR2-EYFP virus into the SCN; each image was obtained from a different animal. Dashed lines denote SCN and the fiber tract. Scale bar represents 100 µm.

(B) Representative short-time Fourier transform spectrogram of EEG activity (above) and plot of EMG amplitude (below) across 10 mins before ("Pre"), 10 mins during ("Stim"), and 40 mins after ("Post") 10 Hz optogenetic stimulation of SCN region, from an animal recorded between ZT2 and ZT6.

(C) Average time (in minutes) for animals to return to baseline (see Methods) following the termination of optogenetic activation of SCN^{mWAKE} neurons (n=4). Data were collected between ZT2 and ZT6.

(D) Representative short-time Fourier transform spectrogram of EEG activity (above) and plot of EMG amplitude (below) across 10 mins before ("Pre"), 10 mins during ("Stim"), and 10 mins after ("Post") 10 Hz optogenetic stimulation of SCN^{mWAKE} neurons in animals injected with AAV-DIO-ChR2-EYFP virus into the SCN (ChR2 (SCN), n=4). Power density is depicted using a color scale and is decomposed by frequency on the y-axis and time on the x-axis. Data were collected between ZT14 and ZT18.

(E) Wakefulness plotted as % time in 5 min bins. Optogenetic stimulation indicated by light blue box. % wakefulness for the 10 mins before, during, and after optogenetic stimulation of SCN^{mWAKE} neurons in mice described in (**D**).

(F) Average wakefulness as % time over the 10 mins before ("Pre"), 10 mins during ("Stim"), and 10 mins after ("Post") optogenetic stimulation of SCN^{mWAKE} neurons in animals injected with AAV-DIO-ChR2-EYFP virus into the SCN described in (**D** and **E**). One-way ANOVA with post-hoc Tukey. $^{*}p < 0.05$.

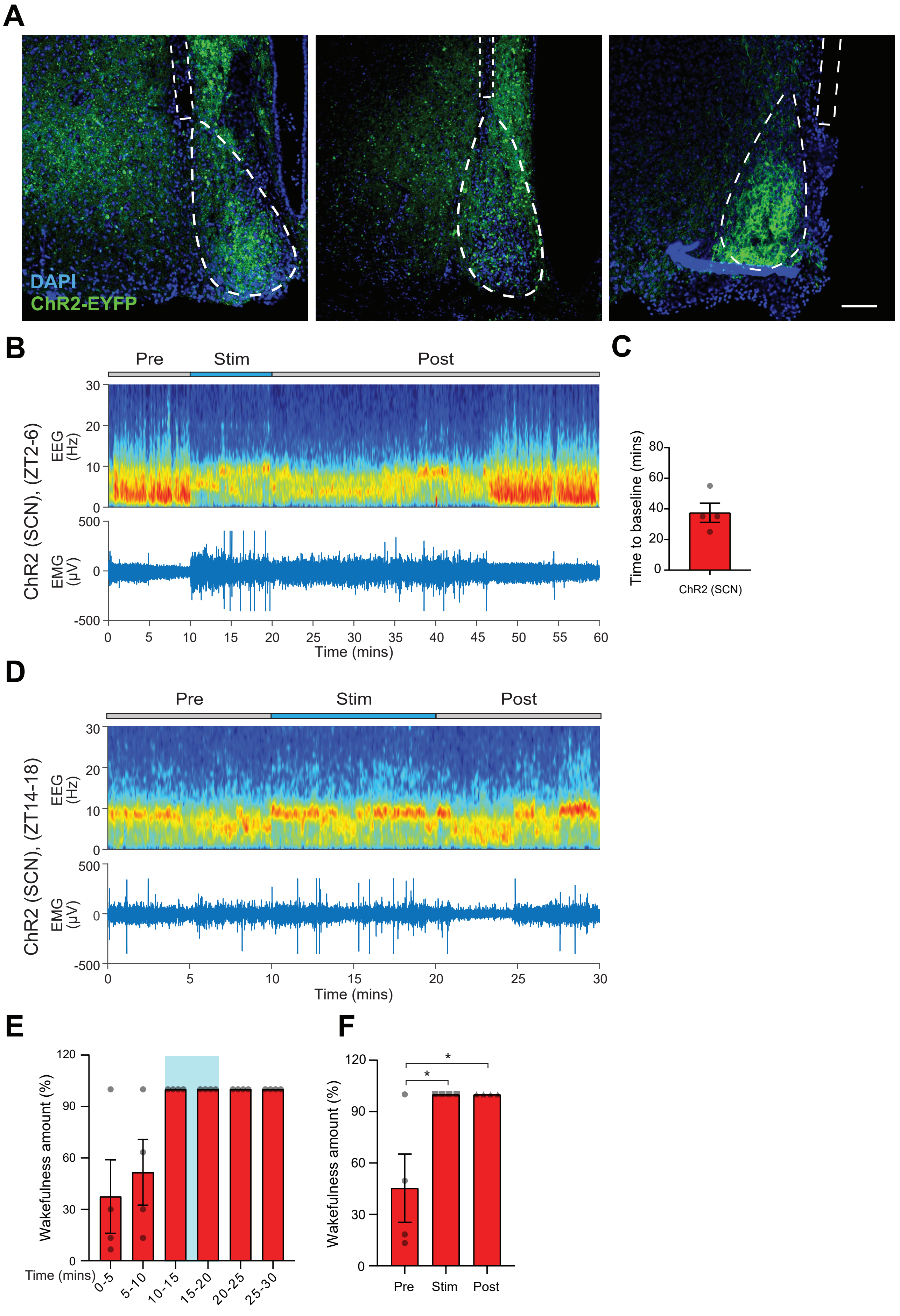
A
DAPI
ChR2-EYFP
B
Pre
Stim
Post
ChR2 (SCN), (ZT2-6)
EEG (Hz)
EMG (µV)
Time (mins)
C
Time to baseline (mins)
ChR2 (SCN)
D
Pre
Stim
Post
ChR2 (SCN), (ZT14-18)
EEG (Hz)
EMG (µV)
Time (mins)
E
Wakefulness amount (%)
Time (mins)
0-5
5-10
10-15
15-20
20-25
25-30
F
Wakefulness amount (%)
Pre
Stim
Post

Figure S2

Figure S3. Validation of conditional knockout of mWAKE in the SCN, related to Fig.3.
(A) Confocal images showing native tdTomato fluorescence and DAPI staining in the SCN of *mWake*$^{(flox/flox)}$ mice following bilateral injection of AAV-Cre-P2A-tdTomato virus into the SCN; each image was obtained from a different animal. Scale bar represents 100 μm.
(B, C) Representative confocal images (**B**) and average *mWake* RNAscope signal intensity (**C**) with tdTomato immunostaining (red), *mWake* (gray) probe, DAPI staining (blue), and merged channels in the SCN of *mWake*$^{(flox/flox)}$ mice injected with AAV-tdTomato (tdTomato, n=5, above) or AAV-Cre-P2A-tdTomato (Cre-tdTomato, n=5, below) virus into the SCN. Scale bar represents 50 μm. Unpaired Student's t-test. $^{***}p < 0.001$.

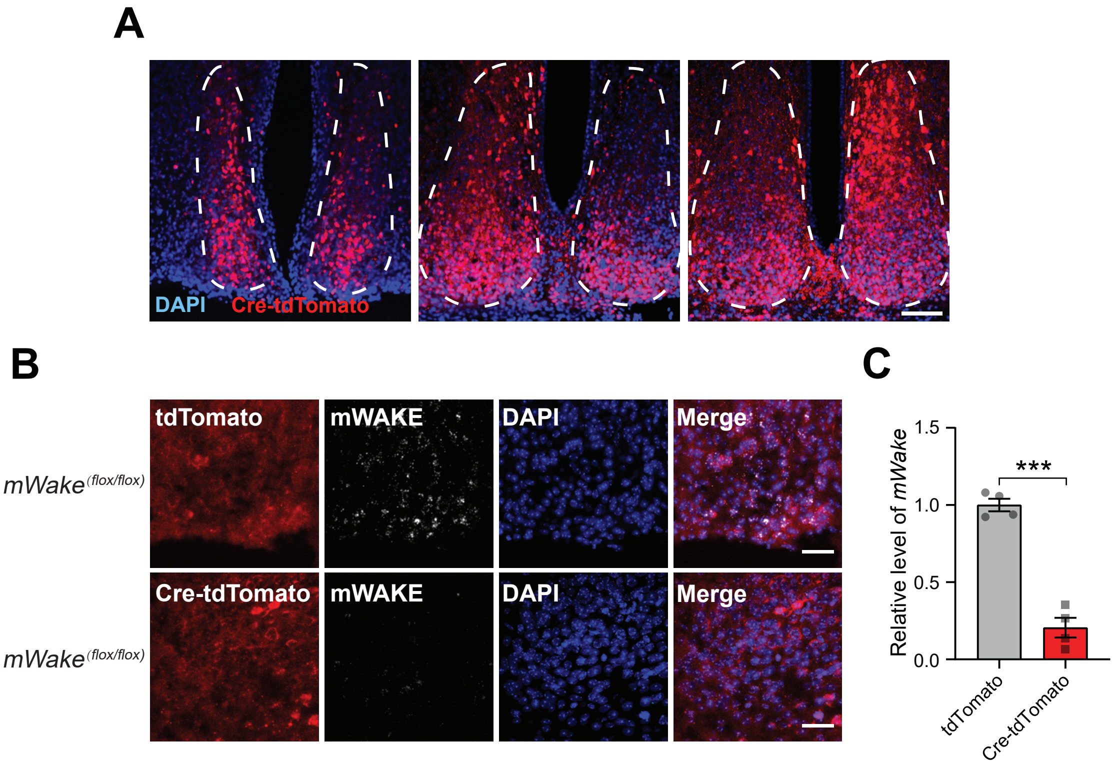
A
DAPI
Cre-tdTomato
B
tdTomato
mWAKE
DAPI
Merge
mWake(flox/flox)
Cre-tdTomato
mWAKE
DAPI
Merge
mWake(flox/flox)
C
Relative level of mWake
1.5
1.0
0.5
0.0
***
tdTomato
Cre-tdTomato

Figure S3

Figure S4. Additional data related to Figure 3.

(A-C) Behavioral state (Wakefulness (**A**), NREM (**B**), REM (**C**)) determined by EEG recordings (% per 2 hr bin) for *mWake*$^{(flox/flox)}$ animals injected with AAV-Cre-P2A-tdTomato (Cre, n=4, red) or AAV-tdTomato (tdTomato, n=4, gray) under LD condition. Yellow and black boxes indicate light and dark periods, respectively.

(D-F) Behavioral state (Wakefulness (**D**), NREM (**E**), REM (**F**)) determined by EEG recordings (% per 12 hr bin) for animals described in (**A-C**). Two-way ANOVA with post-hoc Sidak. $^{**}p < 0.01$.

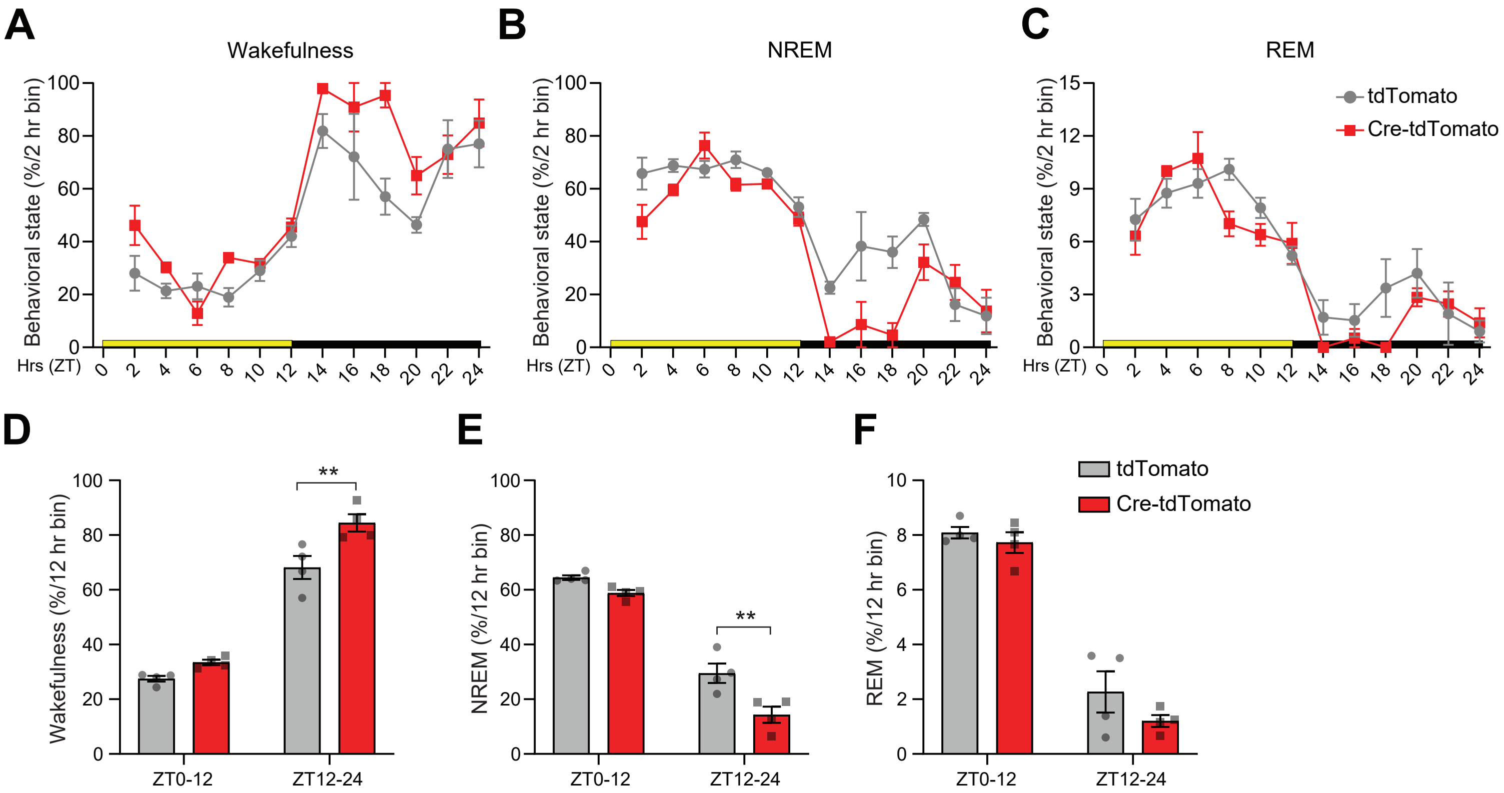

A
Wakefulness
B
NREM
C
REM
Behavioral state (%/2 hr bin)
Hrs (ZT)
0 2 4 6 8 10 12 14 16 18 20 22 24
tdTomato
Cre-tdTomato
D
Wakefulness (%/12 hr bin)
**
ZT0-12
ZT12-24
E
NREM (%/12 hr bin)
**
F
REM (%/12 hr bin)
tdTomato
Cre-tdTomato

Figure S4

Figure S5. Additional data related to Figure 4.

(A) Representative wheel-running traces of *Wildtype (Wt)* and *mWake* knockout (*mWake*$^{(-/-)}$) littermates. 7 days of LD and 7 days of DD data are shown, with yellow bars indicating 12 hr light periods. X-axis represents time in hrs.

(B) Representative chi-squared periodograms for the mice described in (**A**).

(C and **D)** Average period length (**C**) and circadian rhythm amplitude (**D**) for animals described in (**A**) and (**B**) (*Wt*, n=12, gray; *mWake*$^{(-/-)}$, n=6, blue). Unpaired Student's t-test.

(E) Representative confocal images of native tdTomato fluorescence and PER2 immunostaining in the SCN region at CT1 vs CT13 in *mWake*$^{(flox/flox)}$ animals with either AAV-tdTomato (tdTomato, left) or AAV-Cre-P2A-tdTomato (Cre-tdTomato, right) injected bilaterally into the SCN. Merged channels are also shown. Scale bar represents 50 µm.

(F) Relative levels of PER2 intensity in the SCN at CT1 vs CT13 in *mWake*$^{(flox/flox)}$ mice injected with either AAV-tdTomato (tdTomato, gray, CT1, n=3; CT13, n=3) or AAV-Cre-P2A-tdTomato (Cre-tdTomato, red, CT1, n=4; CT13, n=4) injected bilaterally into the SCN. Data represented as fold-change relative to the signal for control animals under CT1 condition. Two-way ANOVA with post-hoc Sidak. $^{**}p < 0.01$.

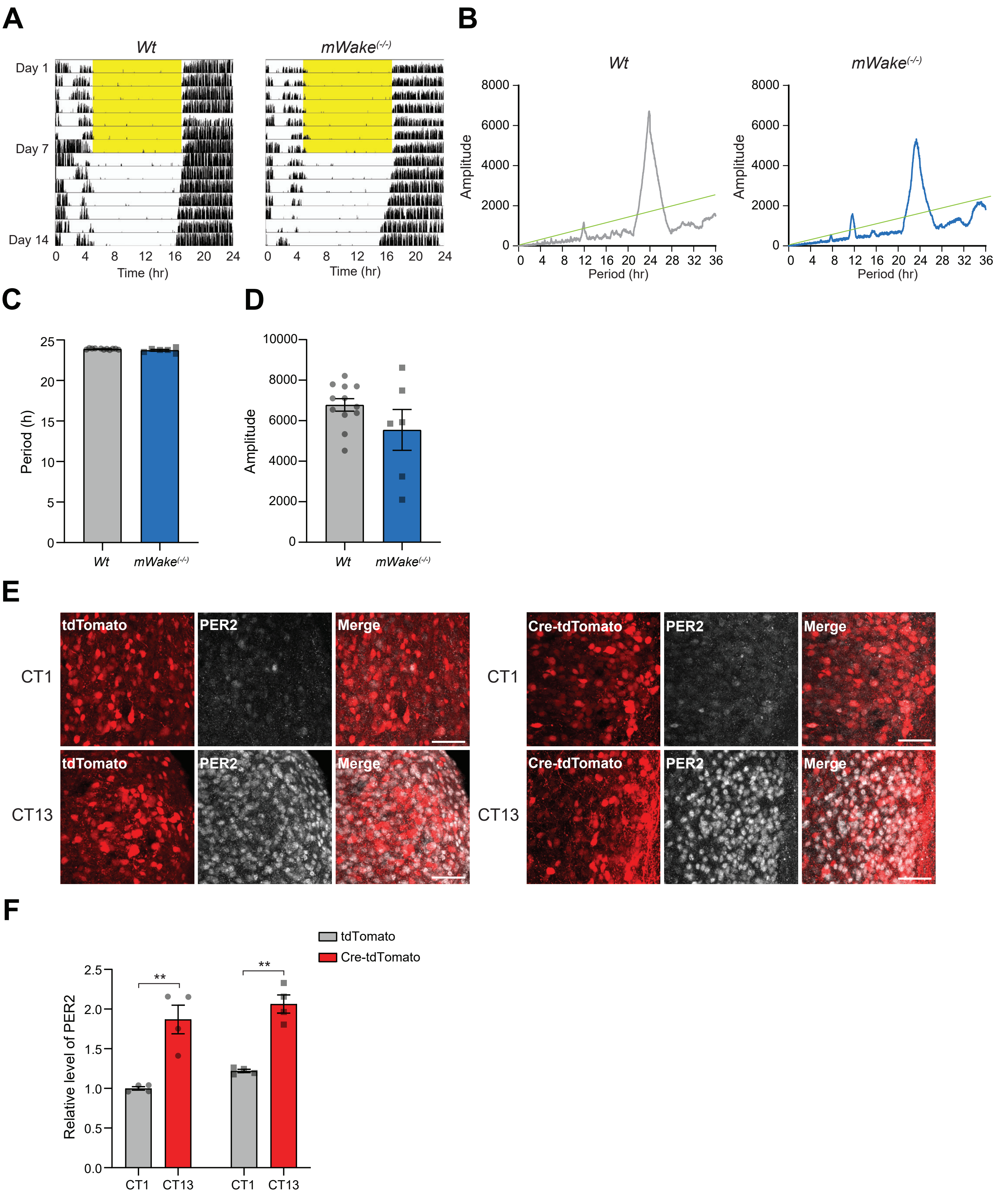

A
Wt
mWake(-/-)
Day 1
Day 7
Day 14
0 4 8 12 16 20 24
Time (hr)
B
Wt
mWake(-/-)
Amplitude
0 2000 4000 6000 8000
0 4 8 12 16 20 24 28 32 36
Period (hr)
C
Period (h)
0 5 10 15 20 25
Wt
mWake(-/-)
D
Amplitude
0 2000 4000 6000 8000 10000
Wt
mWake(-/-)
E
tdTomato
PER2
Merge
Cre-tdTomato
CT1
CT13
F
tdTomato
Cre-tdTomato
Relative level of PER2
0.0 0.5 1.0 1.5 2.0 2.5
**
CT1 CT13 CT1 CT13

Figure S5

Figure S6. Additional data related to Figure 5.

(A) Confocal images showing native EYFP fluorescence and DAPI staining in the SCN of *mWake*$^{(Cre/+)}$ mice bilaterally injected with AAV-DIO-Clock-DN-P2A-EYFP; each image was obtained from a different animal. Dashed lines outline SCN. Scale bar represents 100 μm.

(B-D) Behavioral state (Wakefulness (**B**), NREM (**C**), REM (**D**)) determined by EEG recordings (% per 2 hr bin) for *mWake*$^{(Cre/+)}$ animals injected with AAV-DIO-Clock-DN-EYFP (Clock-DN, green) (n=4) vs AAV-DIO-EYFP (EYFP, gray) (n=5) under LD condition. Yellow and black boxes indicate light and dark periods, respectively.

(E-G) Behavioral state (Wakefulness (**E**), NREM (**F**), REM (**G**)) determined by EEG recordings (% per 12 hr bin) for animals described in (**B-D**). Two-way ANOVA with post-hoc Sidak. $^{*}p < 0.05$.

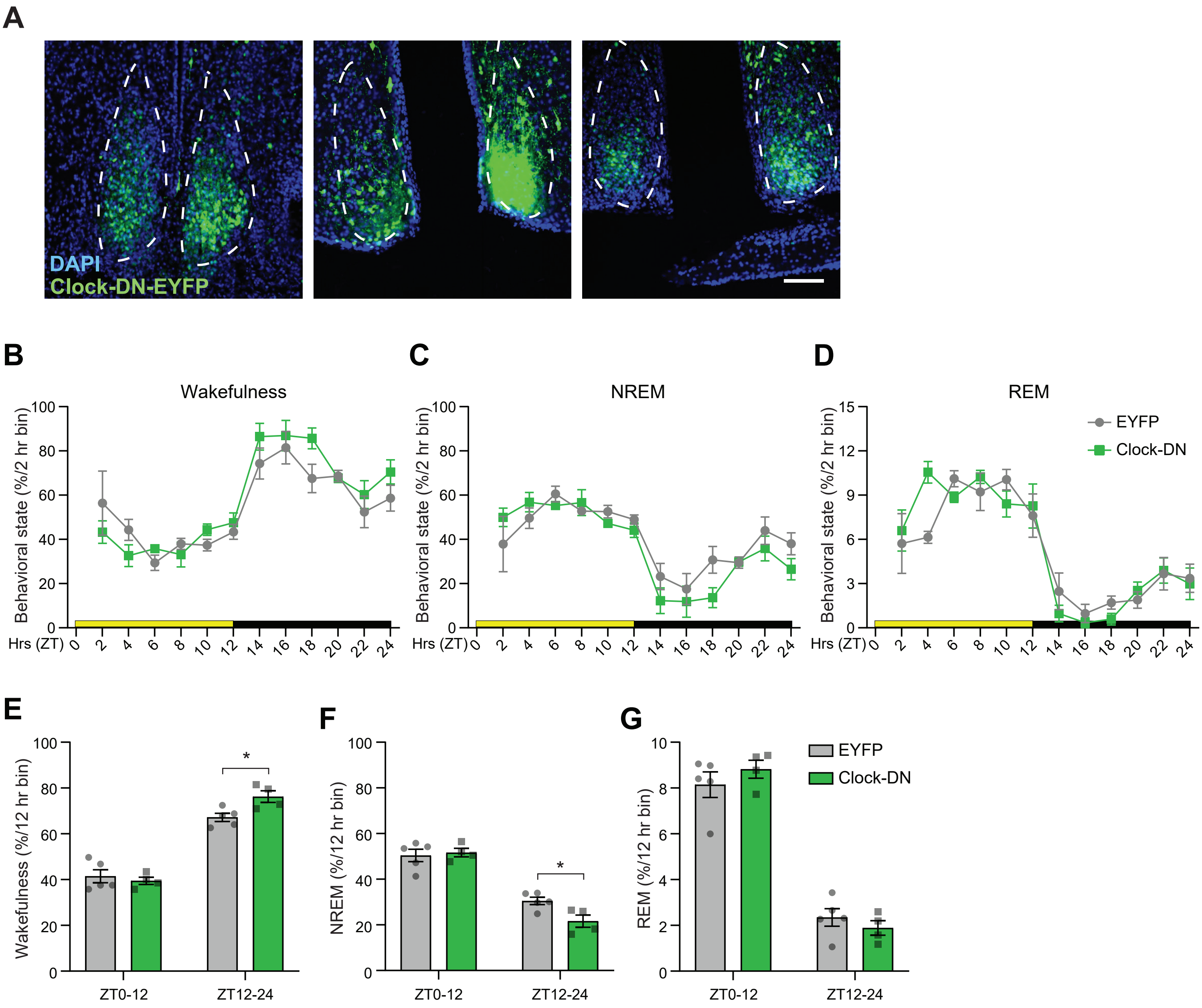

A
DAPI
Clock-DN-EYFP
B
Wakefulness
Behavioral state (%/2 hr bin)
Hrs (ZT)
C
NREM
Behavioral state (%/2 hr bin)
Hrs (ZT)
D
REM
Behavioral state (%/2 hr bin)
Hrs (ZT)
EYFP
Clock-DN
E
Wakefulness (%/12 hr bin)
ZT0-12
ZT12-24
F
NREM (%/12 hr bin)
ZT0-12
ZT12-24
G
REM (%/12 hr bin)
EYFP
Clock-DN
ZT0-12
ZT12-24

Figure S6

Figure S7. Validation of the AAV-DIO-Clock-DN tool in the SCN, related to Fig. 5.

(A) Representative confocal images of EYP native fluorescence, tdTomato and PER2 immunostaining in the SCN region at CT1 vs CT13 in *mWake*$^{(Cre/+)}$ animals with either AAV-DIO-EYFP (EYFP, top) or AAV-DIO-Clock-DN-P2A-EYFP (Clock-DN, bottom) injected into the SCN. Merged channels are also shown. Scale bar represents 50 μm.

(B, C) Relative levels of PER2 intensity in mWAKE^{+} (red) and mWAKE^{-} (gray) cells in the SCN at CT1 vs CT13 from *mWake*$^{(Cre/+)}$ mice injected with either AAV-DIO-EYFP (EYFP, top, CT1, n=3; CT13, n=3) (**B**) or AAV-DIO-Clock-DN-P2A-EYFP (Clock-DN, bottom, CT1, n=4; CT13, n=4) (**C**) injected into the SCN. Data represented as fold-change relative to the signal for control animals under CT1 condition. Two-way ANOVA with post-hoc Sidak. $^{*}p < 0.05$, $^{**}p < 0.01$.

(D) Representative wheel-running traces of *mWake*$^{(Cre/+)}$ animals injected with AAV-DIO-EYFP (EYFP, left) vs AAV-DIO-Clock-DN-EYFP (Clock-DN, right) virus into the SCN. 7 days of LD and 7 days of DD data are shown, with yellow bars indicating 12 hr light periods. X-axis represents time in hrs.

(E) Representative chi-squared periodograms for the mice described in (**D**).

(F and G) Average period length (**F**) and circadian rhythm amplitude (**G**) for the “EYFP” (gray) and “Clock-DN” (green) animals described in (**D**). Unpaired Student's t-test.

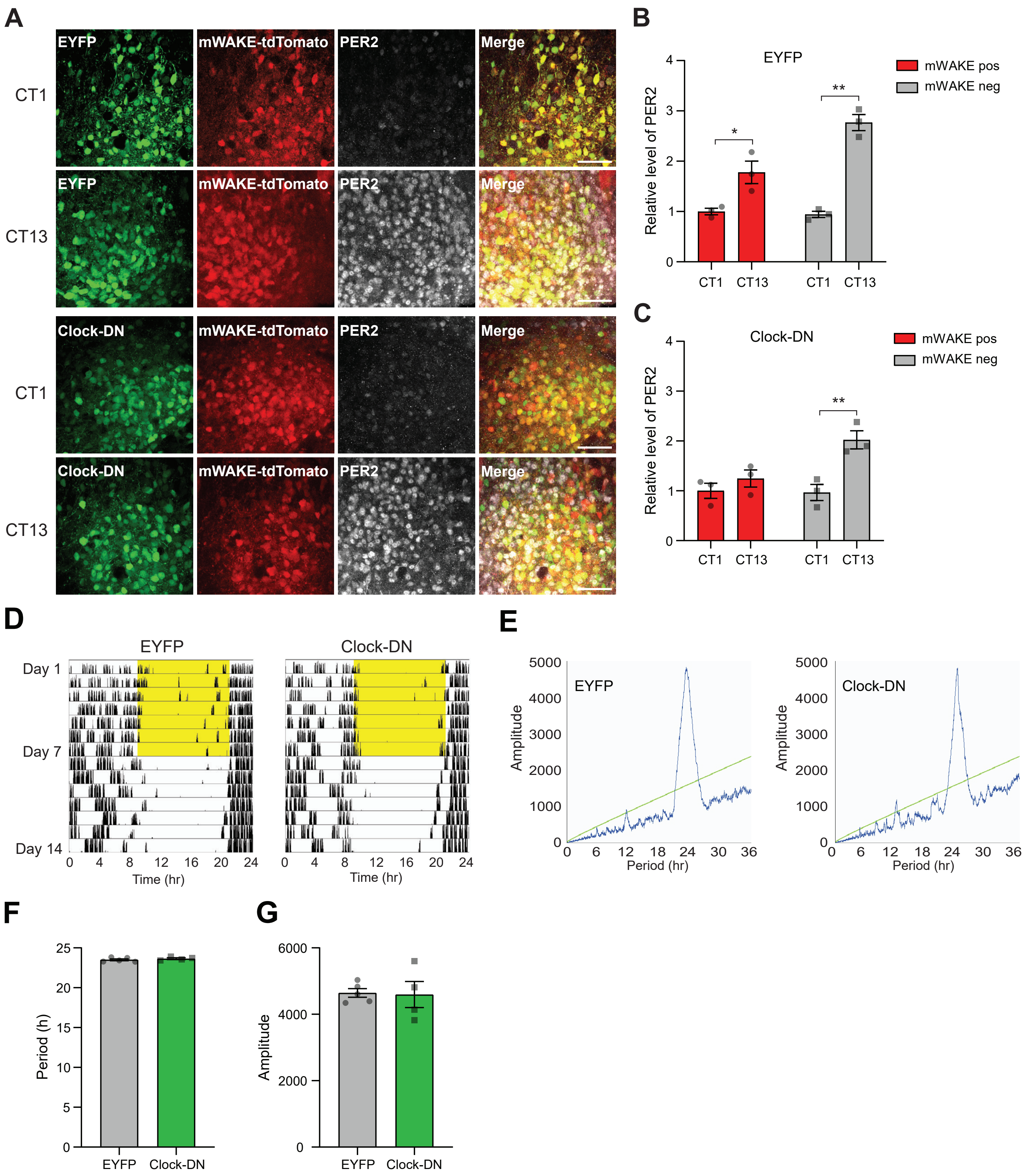
A
EYFP
mWAKE-tdTomato
PER2
Merge
CT1
CT13
Clock-DN
B
EYFP
mWAKE pos
mWAKE neg
Relative level of PER2
CT1
CT13
C
Clock-DN
D
Day 1
Day 7
Day 14
Time (hr)
E
Amplitude
Period (hr)
F
Period (h)
G
Amplitude

Figure S7

Figure S8. Additional data related to Figure 7.

(A) Confocal images showing native EYFP fluorescence, DAPI staining, and optical fiber placement in the SPZ of *mWake*$^{(Cre/+)}$ mice injected with AAV-DIO-ChR2-EYFP in the SCN; each image was obtained from a different animal. Dashed lines outline SPZ and optic fiber tract. Scale bar represents 100 µm.

(B) Representative short-time Fourier transform spectrogram of EEG activity (above) and plot of EMG amplitude (below) across 10 mins before (“Pre”), 10 mins during (“Stim”), and 40 mins after (“Post”) 10 Hz optogenetic stimulation of SPZ region from an animal recorded between ZT2 and ZT6.

(C) Average time (in minutes) for animals to return to baseline (see Methods) following the termination of optogenetic activation of SCN^{mWAKE}→SPZ circuit (n=4). Data were collected between ZT2 and ZT6.

(D) Representative locomotor activity tracks for the 10 min periods before (“Pre”), during (“Stim”), and after (“Post”) optogenetic activation of SCN^{mWAKE} neurons or the downstream SPZ region from an animal recorded between ZT14 and ZT18.

(E) Average speed (m/sec) plotted per 1 min bins for optogenetic activation of SCN^{mWAKE} neurons (n=4, red) or SCN^{mWAKE}→SPZ projections (n=4, purple). Period of optogenetic stimulation indicated by the light blue box; data were collected between ZT14 and ZT18. Shading denotes SEM. Two-way ANOVA with post-hoc Sidak.

(F) Mean speed (m/s) for the 10 min period before (“Pre”), during (“Stim”), or after (“Post”) optogenetic stimulation of SCN^{mWAKE} neurons (n=4, red) or SCN^{mWAKE}→SPZ projections (n=4, purple); data are from the same animals as in E, collected between ZT14 and ZT18. Two-way ANOVA with post-hoc Sidak. $^{***}p < 0.001$.

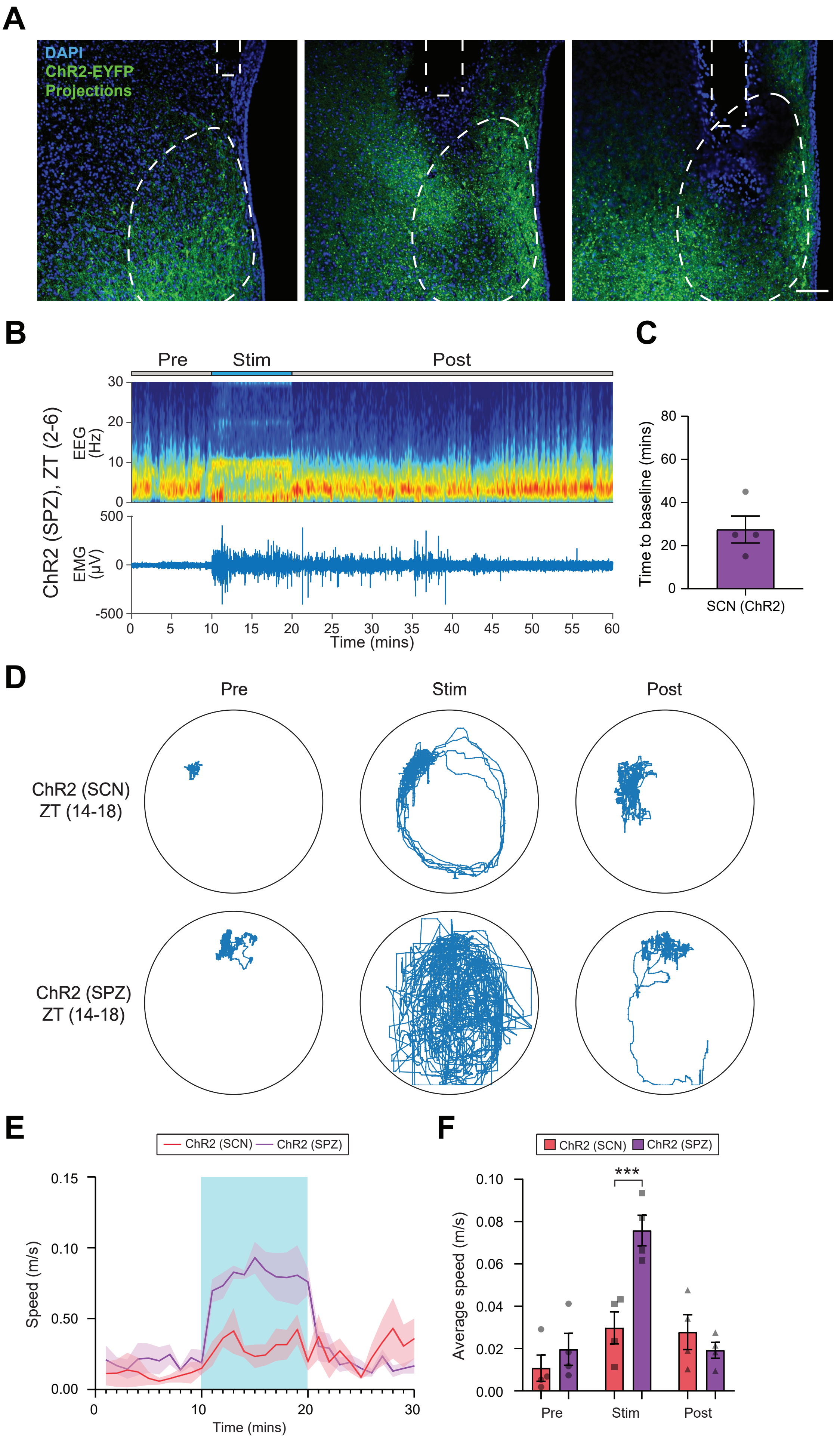
A
DAPI
ChR2-EYFP
Projections
B
Pre
Stim
Post
ChR2 (SPZ), ZT (2-6)
EEG (Hz)
EMG (µV)
Time (mins)
C
Time to baseline (mins)
SCN (ChR2)
D
Pre
Stim
Post
ChR2 (SCN) ZT (14-18)
ChR2 (SPZ) ZT (14-18)
E
ChR2 (SCN)
ChR2 (SPZ)
Speed (m/s)
Time (mins)
F
ChR2 (SCN)
ChR2 (SPZ)
***
Average speed (m/s)
Pre
Stim
Post

Figure S8

Table S1. Full statistical details.

Table S2. Details of stereotaxic coordinates and viruses injected.

Video S1. Representative video showing optogenetic activation of SCN^{mWAKE} neurons in a mouse injected with AAV-DIO-ChR2-EYFP virus into the SCN, collected between ZT2-ZT6.

Video S2. Representative video showing optogenetic activation of SPZ region in a mouse injected with AAV-DIO-ChR2-EYFP virus into the SCN, collected between ZT2-ZT6.

Video S3. Representative video showing optogenetic activation of SCN^{mWAKE} neurons in a mouse injected with AAV-DIO-ChR2-EYFP virus into the SCN, collected between ZT14-ZT18.

Video S4. Representative video showing optogenetic activation of SPZ region in a mouse injected with AAV-DIO-ChR2-EYFP virus into the SCN, collected between ZT14-ZT18.

Table S2. Stereotaxic coordinates and viruses injected

Location	Coordinates (mm)	Virus (Source)	Use	Vol.	Laterality	Titer
**SCN**	AP:-0.4, ML:+0.18, DV:-5.80	AAV9-EF1α-DIO-EYFP (Addgene# 27056-AAV9) "AAV-DIO-EYFP"	Control	100nl	uni-	2.5×10^{13} vg/mL
	AP:-0.4, ML:+0.18, DV:-5.80	AAV9-EF1α-double floxed-hChR2(H134R)-EYFP (Addgene# 20298-AAV9) "AAV-DIO-ChR2-EYFP"	Optogenetic activation	100nl	uni-	2.1×10^{13} vg/mL
	AP:-0.4, ML: +/-0.18, DV:-5.80	AAV5-phSyn1(S)-tdTomato-WPRE (51506-AAV5) "AAV-tdTomato"	Control	50nl	Bi-	2.3×10^{13} vg/mL
	AP:-0.4, ML: +/-0.18, DV:-5.80	AAV2-hSyn-Cre-P2A-dTomato (Addgene# 107738-AAV2) "AAV-Cre-tdTomato"	Conditional knockout	50nl	Bi-	2.5×10^{13} vg/mL
	AP:-0.4, ML:+/-0.18, DV:-5.80	AAV9-EF1α-DIO-EYFP (Addgene# 27056-AAV9) "AAV-DIO-EYFP"	Control	100nl	Bi-	2.5×10^{13} vg/mL
	AP:-0.4, ML:+/-0.18, DV:-5.80	AAV2/9-EF1α-DIO-Clock-DN-EYFP (BioHippo) "AAV-DIO-Clock-DN-EYFP"	Cre-on Clock-DN	100nl	Bi-	1.1×10^{13} vg/mL
	AP:-0.4, ML:+0.18, DV:-5.75		Optic fiber implantation		uni-	
**SPZ**	AP:-0.7, ML:+0.15, DV:-5.3		Optic fiber implantation		uni-	
